# Coexistence of AI Nurse and AI-Simulated Patient in Nursing Education Using a Bidirectional Fine-Tuning Pipeline: Development and Content Analysis Study

**DOI:** 10.2196/100778

**Published:** 2026-11-06

**Authors:** Takeshi Koike, Yukie Majima

**Affiliations:** 1 Faculty of Nursing, Seirei Christopher University, 3453 Mikatahara-cho, Chuo-ku, Hamamatsu, Japan, 81 053-439-1401; 2 Graduate School of Informatics, Osaka Metropolitan University, Osaka, Japan

**Keywords:** nursing education, large language model, simulated patient, AI nurse, LoRA fine-tuning, bidirectional fine-tuning, functional asymmetry, simulation ecosystem, Berelson content analysis, disaster preparedness education, low-rank adaptation

## Abstract

**Background:**

Simulated patient systems are essential in nursing education but face actor scarcity, instructor burden, cost, and disrupted continuity where in-person simulation cannot be sustained. Existing large language model (LLM)–based systems address these issues but treat the dialogic partner—the AI nurse—as a static prompt or unspecialized model, compromising data collection quality and pedagogical applicability.

**Objective:**

We report (1) the construction of 2 specialized fine-tuned models, NURSE-ONE (AI nurse) and PATIENT-ONE (AI-simulated patient), via a 5-step bidirectional fine-tuning pipeline; (2) their evaluation using Berelson content analysis validated by independent human coders; (3) an exploratory third-party evaluation by nursing faculty; and (4) the design of a coexistence simulation ecosystem supporting 3 operating modes, including offline disaster preparedness applicability.

**Methods:**

On a single consumer-grade graphics processing unit (NVIDIA RTX 4090; 24 GB video random access memory), the multimodal base model gemma4-E4B-it was fine-tuned via low-rank adaptation in five steps: (1) a 251-scenario base from 29 institutionally authored nursing care plans and 20 items from the 115th Japanese National Nursing Examination; (2) a provisional dialogue system (AutoGen v18, maxmsg=25) to collect training dialogues; (3) NURSE-ONE fine-tuning across 3 training volume conditions (245, 735, and 1225 cases); (4) 4-condition Berelson comparison; and (5) PATIENT-ONE fine-tuning on NURSE-ONE–generated dialogues with a 3-condition comparison. Interrater reliability was confirmed by 2 independent nursing experts (196 blinded utterances, κ=0.770) and corroborated by 2 LLM raters (κ=0.742). Ten nursing faculty evaluated 3 coexistence modes in a working web user interface (21-item rubric; item-level content validity index=1.00).

**Results:**

Four-condition Berelson analysis (*χ*^2^_15_=42.0, *P*<.001) identified the 735-case nurse-side condition (open question rate=21.5%) as NURSE-ONE; information collection rate reached 15.5% (~4× baseline) at 1225 cases. PATIENT-ONE A/B/C analysis (*χ*^2^_10_=11.8, *P*=.30) showed no significant category shift but the lowest response length variability (SD 24.5) and highest information-providing rate (47.9%) at 735 cases, adopted as PATIENT-ONE. The 2 adapters appeared to operate on different axes—qualitative questioning style on the nurse side and quantitative response stability on the patient side—a preliminary observed pattern we term functional asymmetry, pending replication. Third-party evaluation rated the AI-simulated patient, AI nurse, and dyadic interaction at 90.3%, 69.0%, and 84.8%, respectively, with role maintenance at ceiling (mean 5, SD 0). All 3 modes met the prespecified 3-second latency threshold.

**Conclusions:**

A 5-step bidirectional fine-tuning pipeline produced 2 specialized models whose joint deployment constitutes a design pattern for nursing education simulation. The preliminarily observed functional asymmetry between qualitative nurse-side variation and quantitative patient-side stabilization appears to underwrite the system’s coherence, supporting 3 operating modes on consumer-grade hardware and deployable offline in disaster preparedness contexts. Third-party evaluation confirmed exploratory educational applicability, with all evaluators rating the system as usable, providing a foundation for future learner outcome studies.

## Introduction

### Simulation-Based Learning and the Limitations of Standardized Patients

Simulation-based learning is a cornerstone of contemporary nursing education, with systematic reviews and meta-analyses consistently demonstrating its effectiveness in developing clinical reasoning, communication, and procedural skills [1,2]. Standardized patients remain the gold standard for communication-focused simulation, supported by formalized standards of best practice [3]. However, the standardized patient modality faces well-documented structural challenges: the scarcity and high training cost of qualified actors, educator burden, inequitable opportunities across institutions, and—as illustrated by both pandemic disruption and armed conflict environments—the inability to maintain in-person continuity [4-6]. Although standardization and dissemination of standardized patient training programs have progressed in Japan and internationally [3,7], the ongoing cost of actor training and maintenance remains substantial. These pressures motivate a growing search for scalable, reproducible alternatives that remain operable when in-person infrastructure is degraded.

### Large Language Model–Based Simulated Patients: Rapid Expansion and a Structural Blind Spot

Since 2023, large language models (LLMs) have driven a rapid expansion of computational simulated patient research. Foundational work demonstrated that general-purpose LLMs encode clinically relevant knowledge [8], and subsequent studies showed that LLM-based agents can play standardized patient roles in dialogue: Du et al [9] proposed an agent coevolution framework, Schmidgall et al [10] benchmarked clinical AI in simulated environments (AgentClinic), Kyung et al [11] introduced a persona-driven simulator (PatientSim), Jiang et al [12] developed a multistage role-playing framework, and Yu et al [13] reported that LLM-based simulated patients can transform medical education at scale. The medical dialogue line beginning with ChatDoctor [14] has extended to role-play strengthening approaches such as RoleplayLLM [15]. Comprehensive systematic reviews of LLM-based virtual patients are now appearing [16,17], and conversational diagnostic AI is advancing rapidly [18,19]. Earlier reviews of virtual patient simulations in health professions education [20] further establish the field’s pedagogical foundations.

Despite this progress, virtually all existing systems focus exclusively on the patient side of the dyad. A targeted survey of the 7 principal LLM-based simulated patient projects [9-15] revealed that 5 had not performed any fine-tuning, all relied on models with 70 B+ parameters or commercial APIs (eg, GPT-4), and none had specialized the nurse or clinician role. Available dialogue datasets [21,22] are insufficient in scale and curation for nurse-side fine-tuning. The bidirectional fine-tuning of both nurse and patient roles in nursing education thus remains unexplored territory.

### The Overlooked Partner: Bidirectional Fine-Tuning as a Development Strategy

If the nurse role is the data source as well as the dialogic partner, the unspecialized nurse becomes a structural bottleneck. When a general-purpose LLM is asked to play the nurse role during training data generation, observed problems include shallow questioning, missed assessment cues, lack of empathic responses, and dialogue degeneration phenomena such as the “farewell loop.” Patient-side data collected from such dialogues inherit these limitations.

We therefore adopted a bidirectional fine-tuning strategy: NURSE-ONE was fine-tuned first; the resulting model was then used to generate dialogue data that became the training corpus for PATIENT-ONE. The pipeline comprised five sequential steps: (1) construction of a scenario base from institutionally authored standard nursing care plans and national examination items; (2) construction of a provisional dialogue system to collect training-eligible dialogues; (3) NURSE-ONE fine-tuning with multiple training volume conditions; (4) 4-condition Berelson comparison to select the production NURSE-ONE; (5) PATIENT-ONE fine-tuning on NURSE-ONE–generated dialogues followed by A/B/C Berelson comparison. The complete pipeline yields 2 production models: NURSE-ONE (the 735-case nurse-side condition, open question [OQ] rate=21.5%) and PATIENT-ONE (the 735-case patient-side condition, information collection [IN] rate=47.9%, SD 24.5). The naming convention designated the production models; intermediate prototype versions were referred to as “prototype nurse 1/2/3” and “prototype patient 1/2.”

### From Bidirectional Construction to Coexistence Application

A natural question follows from this construction: when NURSE-ONE and PATIENT-ONE are operated together—rather than separately—what additional value emerges? The 2 models share layer 1 (base model) and layer 3 (scenario prompt) but specialize on distinct layer 2 axes (nurse side: questioning style; patient side: response stability). We argue that this asymmetric specialization is not redundant but complementary: their coexistence may produce a balance between pedagogical diversity (nurse) and learning condition reproducibility (patient).

We further argue that a coexistence configuration enables a small set of pedagogically distinct operating modes that single-agent systems cannot easily realize: (1) AI-AI demonstration, in which both agents converse and the learner observes; (2) student-as-nurse, in which the learner replaces NURSE-ONE and engages with PATIENT-ONE while NURSE-ONE provides reference behavior or post hoc feedback; and (3) student-as-observer, in which the learner annotates or evaluates AI-AI dialogue. These correspond to established pedagogical roles—observation, deliberate practice, and reflective evaluation. Critically, because the system runs entirely on a single consumer-grade graphics processing unit (GPU) without cloud dependence, all 3 modes remain operable in disaster preparedness contexts where cloud-dependent alternatives are not.

### Research Questions

The following research questions (RQs) were formulated:

1. RQ1: can a 5-step bidirectional fine-tuning pipeline specialize a lightweight base LLM into a nurse role and a patient role with measurable, complementary effects on dialogic behavior?
2. RQ2: does increasing training data volume on the nurse side improve Berelson dialogic indicators monotonically and does training data variation on the patient side affect dialogue style and response length?
3. RQ3: under coexistence (NURSE-ONE × PATIENT-ONE), can 3 pedagogically distinct operating modes be instantiated within practical response time constraints (≤3 seconds per turn)?
4. RQ4: what design principles for AI-driven nursing simulation ecosystems can be derived from the preliminarily observed functional asymmetry between nurse-side and patient-side fine-tuning, and what implications follow for offline disaster preparedness deployment?

## Methods

### Study Design and Overview of the 5-Step Pipeline

The development followed a 5-step bidirectional fine-tuning pipeline (Figure 1; Multimedia Appendix 1):

1. Scenario base construction: 251 scenarios assembled from 29 institutionally authored standard nursing care plans (7 domains × observation plan/treatment plan/education plan [OP/TP/EP] 3-axis structure) and 20 situational items from the 115th Japanese National Nursing Examination;
2. Provisional dialogue system: AutoGen v18 multiagent dialogue collection with maxmsg=25 (calibrated against established degeneration metrics);
3. NURSE-ONE fine-tuning: 3 prototype nurse models trained on 245/735/1225 cases (preset × 1/× 3/× 5);
4. Four-condition nurse-side Berelson comparison: production NURSE-ONE selected on the basis of open question rate optimization; and
5. PATIENT-ONE fine-tuning and A/B/C Berelson comparison: 2 prototype patient models trained on NURSE-ONE–generated dialogues; production PATIENT-ONE selected.

**Figure 1.**
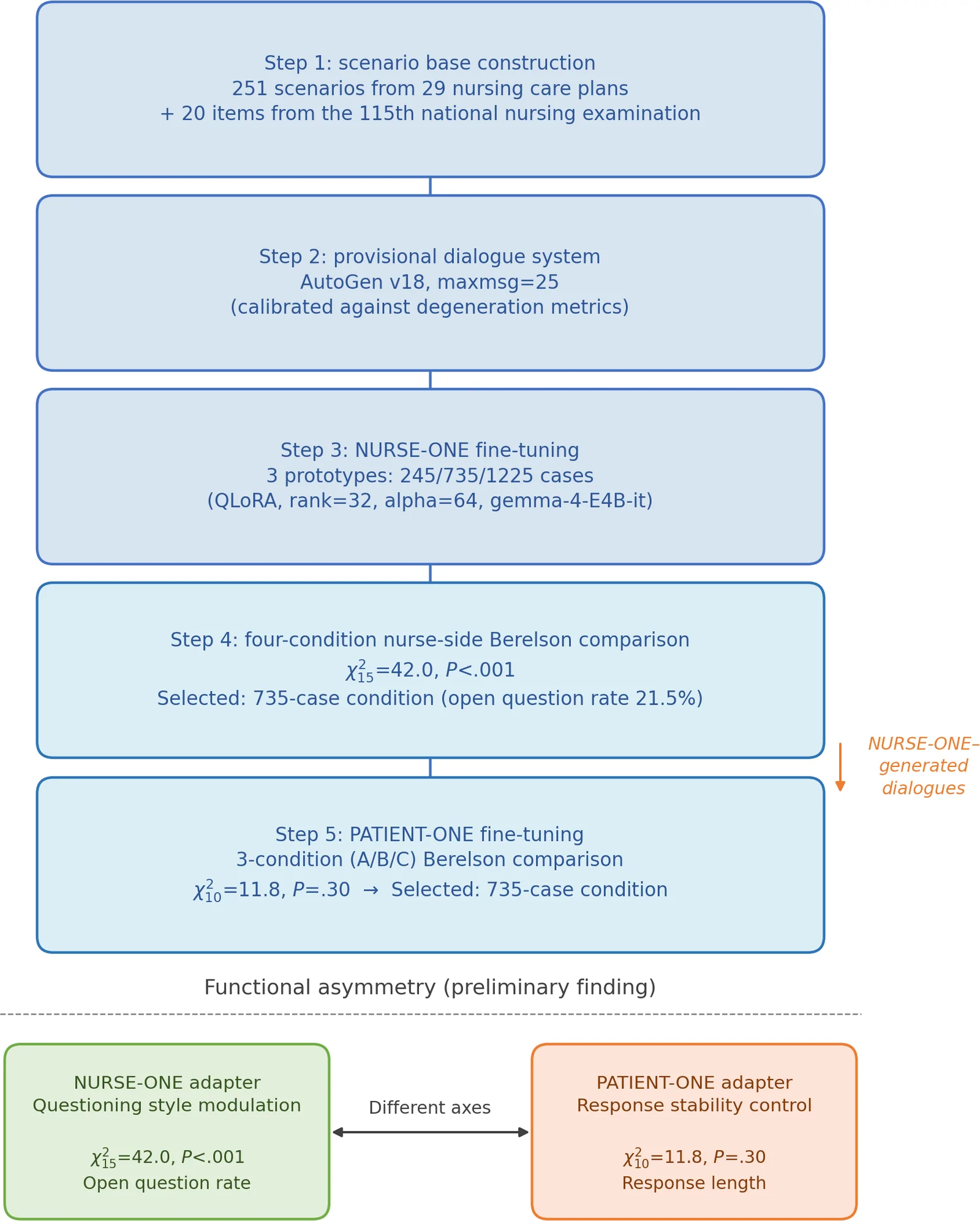
Five-step bidirectional fine-tuning pipeline. The scenario base (step 1) feeds dialogue collection (step 2), whose output trains 3 prototype nurse models (step 3). Berelson content analysis selects the production NURSE-ONE (step 4), which then generates the training corpus for PATIENT-ONE (step 5). QLoRA: quantized low-rank adaptation.

### Development Environment

A single Windows 11 (Microsoft) workstation equipped with an NVIDIA RTX 4090 (24 GB video random access memory [VRAM]) was used. Python (version 3.11; Python Software Foundation) with Unsloth (Unsloth AI), Transformers (Hugging Face), and PEFT (Hugging Face) was used for fine-tuning; llama.cpp (build b8793; ggml.ai) was used for GGUF conversion; Ollama (Ollama Inc) was used for inference serving; and AutoGen (Microsoft) was used for multiagent dialogue collection. All software ran locally without cloud dependence.

### Base Model Selection

Six candidate base models were compared against Japanese-language role-play criteria; 3 passed the practical use thresholds. gemma4-E4B-it was selected for both NURSE-ONE and PATIENT-ONE on the basis of (1) Japanese fluency in role-play, (2) inference speed (1.5‐2.0 seconds per nurse response in chronic obstructive pulmonary disease scenarios on RTX 4090), (3) gemma4-E4B-it × gemma4-E4B-it dyadic dialogue at 2.5 to 2.9 seconds per turn, and (4) preserved multimodality (vision_tower and audio_tower) for future multimodal extension.

### Three-Layer Architecture

Both NURSE-ONE and PATIENT-ONE shared a 3-layer architecture (Figure 2):

- Layer 1—base model: gemma4-E4B-it, identical for both roles
- Layer 2—specialization (low-rank adaptation [LoRA] adapter): role-specific behavioral patterns; nurse-side common questioning patterns and patient-side common response patterns; the LoRA adapter is merged into base weights at deployment
- Layer 3—individualization (scenario prompt): per-case clinical context (timing, vital signs, family composition, and individual situation), drawn from the 251-scenario base; layer 3 is identical in structure for both roles; only the per-case content differs

This design isolated role specialization in layer 2 and enabled a single base model + single scenario substrate to drive both roles—a structural prerequisite for the coexistence configuration described below.

**Figure 2.**
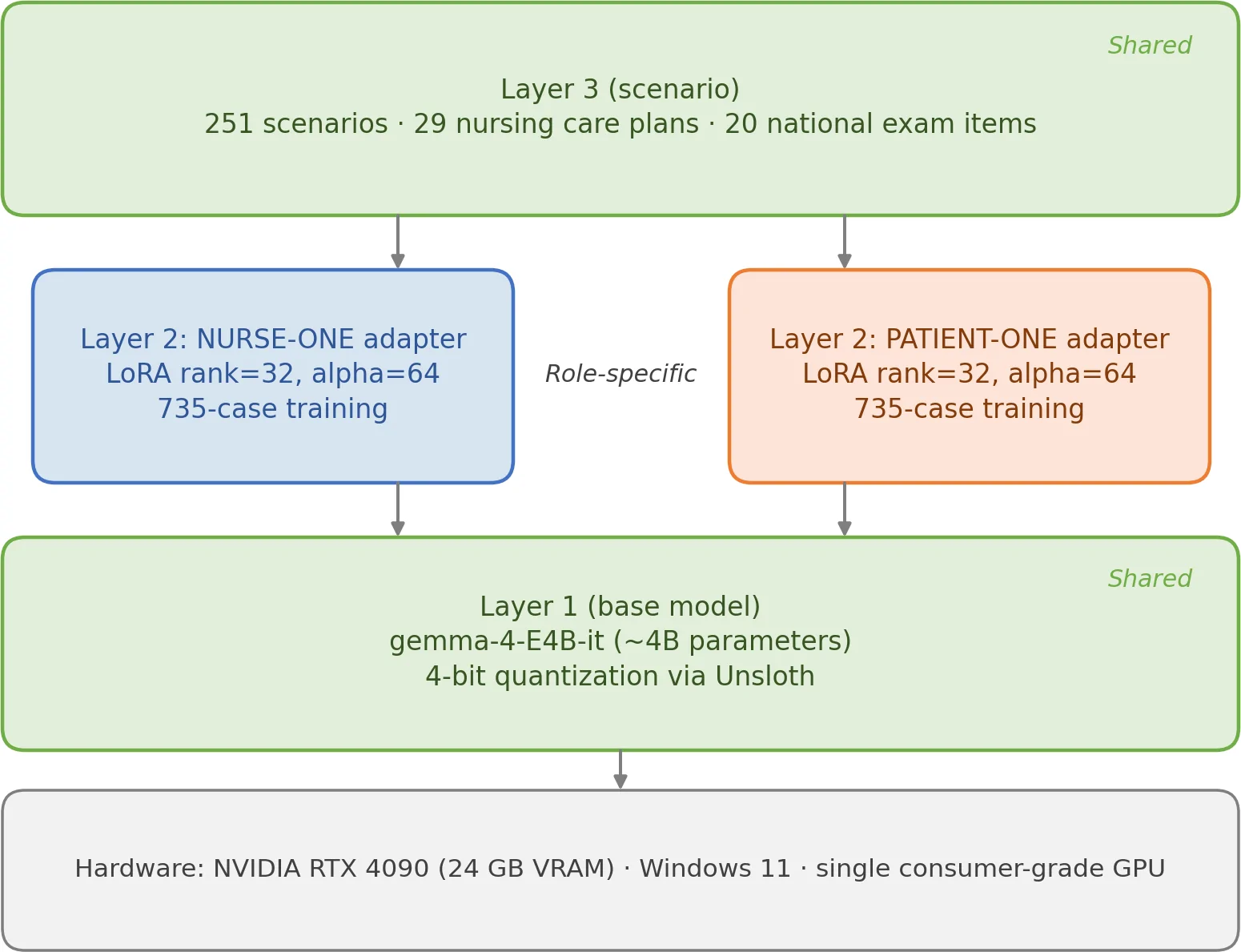
Three-layer architecture shared by NURSE-ONE and PATIENT-ONE. Layer 1 (gemma4-E4B-it base) and layer 3 (scenario prompt) are common; layer 2 carries role-specific LoRA adapters that appear to operate on different axes (see the *Functional Asymmetry Between Nurse-Side and Patient-Side Fine-Tuning* section). GPU: graphics processing unit; LoRA: low-rank adaptation; VRAM: video random access memory.

### Educational Content Base

#### Standard Nursing Care Plans (29 Diseases)

TK authored 29 standard nursing care plans as HTML educational materials over an 11-day period. Existing nursing care plan textbooks are written in expository prose unsuited to LLM scenario prompts, which require a dialogue-driven representation that includes patient-subjective experience, emotion, and disease acceptance stage. The 29 plans cover 7 domains (acute, chronic, geriatric, pediatric, psychiatric, maternal, and home care) and use a 3-axis OP/TP/EP structure. Clinical validity was secured by cross-checking against standard nursing curriculum textbooks and TK’s 17 years of nursing education practice.

#### 115th Japanese National Nursing Examination—Situational Items

All 20 situational items from the 115th Japanese National Nursing Examination (administered February 2026) were reconstructed as simulated patient scenarios. Each item supplied a patient profile (age, sex, diagnosis, and hospitalization context) and a clinical scene. The corresponding correct nursing actions—drawn from a multiexpert-reviewed national licensing examination—were encoded into the nurse-side prompt as response policy. The items covered the following scenarios: adult medical/surgical (8 items), psychiatric (3 items), pediatric (3 items), geriatric/home care (3 items), maternal/obstetric (2 items), and emergency/disaster (1 item). National examination items contributed clinical judgment validity and broadened scenario diversity beyond the standard care plans.

The combined 251-scenario base (Multimedia Appendix 2) is the shared input substrate for both layer 3 prompts and dialogue collection runs. Of these, 245 scenarios were eligible for fine-tuning data generation (6 scenarios with incomplete clinical profiles or ambiguous dialogue end points were excluded from the training pool but retained in the scenario base for coexistence mode evaluation).

### Dialogue Data Collection System

#### Multiagent Pipeline Configuration

AutoGen (Microsoft) was used to construct a multiagent dialogue collection pipeline. The collection system iterated through scenarios, instantiated the nurse-role and patient-role agents per scenario, and recorded multiturn dialogues for downstream fine-tuning.

#### Maxmsg Calibration and Farewell Loop Control

A farewell loop problem—repeated farewell utterances by the nurse-role LLM after assessment completion—was observed in initial collection. To calibrate the per-session message budget (maxmsg), 4 conditions (maxmsg=20/30/40/50) were each run for 100 trials (total 400 trials). Two established degeneration metrics were applied: seq-rep-4 [23] with degeneration threshold >0.3 and Distinct-2 [24] with low-diversity threshold <0.5. Maximum degeneration rate across all conditions was below 1%. An ad hoc keyword frequency × SequenceMatcher metric, also tested, produced up to 62× overestimation of degeneration relative to the established metrics, confirming the appropriateness of the established methodology. On the basis of these results, maxmsg=25 was adopted as the operating value.

### NURSE-ONE Fine-Tuning Design

#### Hybrid Approach Following ChatDoctor

Fine-tuning followed the hybrid approach of ChatDoctor [14]: dialogue data collected by the system described above served as the training corpus, structured according to the 3-layer architecture so that the LoRA adapter encodes nurse-side common questioning patterns rather than scenario-specific knowledge.

#### Training Volume Conditions for the Experience Volume Hypothesis

A central design question was whether training data volume monotonically improves nurse-side dialogic quality—an experience volume hypothesis analogous to Benner’s “novice-to-expert” model in nursing skill development [25] and consistent with reports that accumulated clinical experience significantly improves clinical judgment capability [26]. Three training volumes were systematically varied: prototype nurse 1 was trained on 245 cases (preset × 1), analogous to first-year experience; prototype nurse 2 was trained on 735 cases (preset × 3), analogous to third-year experience; and prototype nurse 3 was trained on 1225 cases (preset × 5), analogous to fifth-year experience.

Final loss values monotonically decreased with volume (2.36 → 1.537 → 0.840), consistent with the experience volume hypothesis at the loss level; whether dialogic quality follows the same trend is reported in the *Results* section.

### Model Merging and Deployment

LoRA adapters were merged into the base weights using parameter-efficient fine-tuning (PEFT). Because gemma4-E4B-it carries vision_tower (vision processing), audio_tower (audio processing), and multi_modal_projector heads, the merge command used exclude_modules to exclude these from the merge, preserving multimodal capacity in the merged checkpoint. Merged models were converted to the GGUF format using llama.cpp (b8793), quantized to q4_k_m (5.07 GB per role), and registered with Ollama for inference serving.

### Berelson Content Analysis

#### Dialogue evaluation

Dialogue evaluation used Berelson content analysis [27]. The unit of analysis was the per-turn nurse utterance (or patient utterance, in patient-side experiments). The 6-category schema covered OQ, closed question (CQ), IN, assessment confirmation (AC), empathic acknowledgment (EM), and other (OT). The schema was applied with role-appropriate interpretations: on the nurse side, IN denotes information collection questions, while on the patient side, IN denotes information-providing utterances; OQ on the patient side is rare and usually represents reciprocal clarification. To avoid cross-role confusion, all numerical IN and OQ values reported below specify whether they are nurse side or patient side.

Because the unit of analysis was the individual utterance, the primary analysis treated each utterance as an observation. We note that utterances were nested within dialogues and scenarios. To assess whether this nesting structure affected the findings, we computed condition-level category proportions aggregated at the scenario level and confirmed that the patterns reported below were consistent with the utterance-level analysis. Scenario-level aggregation did not alter the direction or significance of any comparison.

#### Human Coder Validation

To validate the Berelson classification scheme, 2 nursing experts (both with clinical and teaching experience) independently classified a stratified subsample of utterances drawn from the 4-condition nurse-side experiment. The subsample comprised 200 utterances (50 per condition), selected by stratified random sampling (seed=20260721) that preserved category proportions within ±2% of the population distribution. Utterances were presented in a blinded form: condition labels were stripped and replaced with randomized set identifiers (sets A-D). Coders received the same 6-category definitions and classification rules used in the LLM-assisted classification. Cohen κ was computed on the classifications of the 2 experts (n=196; 4 utterances were excluded due to missing responses from one coder). The interpretation of κ follows Landis and Koch [28]: <0.00, poor; 0.00 to 0.20, slight; 0.21 to 0.40, fair; 0.41 to 0.60, moderate; 0.61 to 0.80, substantial; 0.81 to 1.00, almost perfect.

#### LLM-Assisted Classification

To enable scalable, reproducible classification across the full dataset (>800 nurse-side and 720 patient-side utterances), 2 LLMs—Claude Sonnet 4.5 (primary rater) and Claude Opus 4.6 (secondary rater)—served as automated raters. The category schema and exemplars were specified in a fixed prompt; raters had no access to model identity (condition labels were stripped). This approach follows the emerging practice of LLM-as-rater methodology, whose validity is examined in the *Discussion* section in the light of the human coder validation results.

#### Four-Condition Nurse-Side Comparison (NURSE-ONE Selection)

Four conditions—the gemma4-E4B-it base nurse (control) plus prototype nurses 1/2/3—were compared with 200 utterances per condition. Interrater reliability between the 2 LLM raters was computed as Cohen κ on 100 utterances (seed=42), yielding κ=0.742 (run1 κ=0.736, run2 κ=0.749). Pairwise comparisons used 2-tailed Fisher exact tests at *α*=.05; the 4-condition omnibus statistic was *χ*^2^_15_=42.0 (*P*<.001).

#### Three-Condition Patient-Side Comparison (PATIENT-ONE Selection)

Three conditions—the gemma4-E4B-it base patient (A) plus prototype patients 1 (B; preset × 1) and 2 (C; preset × 3)—were compared with 240 utterances per condition. The same 2-LLM rater configuration was used. Interrater reliability for patient-side classification was κ=0.718 on a condition C sample (n=100, seed=42). Mixed-condition sampling produced κ=0.659; condition C–restricted sampling was adopted because patient-side utterances across conditions exhibited greater surface-level similarity than nurse-side utterances, making cross-condition classification more ambiguous for both human and automated raters. The 3-condition omnibus statistic was *χ*^2^_10_=11.8 (*P*=.30); the only pairwise statistically significant residual was AC in condition B (z=+2.30, *P*=.02).

### Third-Party Expert Evaluation

To assess educational applicability beyond the Berelson content analysis metrics, a third-party evaluation was conducted as part of the companion study [29]. Ten nursing faculty members (teaching experience 3‐20 years) independently evaluated the system via a web-based evaluation platform with a permanent URL. Each evaluator completed two phases: (1) observation of an AI-AI dialogue (mode A: NURSE-ONE × PATIENT-ONE) and (2) direct interaction with PATIENT-ONE in the nurse role (mode B). After each phase, evaluators completed a structured rubric.

The rubric comprised 21 items on a 5-point scale with anchored descriptors, organized into 3 subscales: subscale A (AI nurse performance; 8 items, 40 points; informed by the SEGUE [set the stage, elicit information, give information, understand the patient’s perspective, and end the encounter] framework for medical communication [30]), subscale B (AI-simulated patient performance; 8 items, 40 points; informed by the authenticity constructs of the Maastricht Assessment of Simulated Patients [31]), and subscale C (dyadic interaction quality; 5 items, 25 points). Content validity was established by 3 expert reviewers (nursing education experience 5‐20 years) who rated item relevance on a 4-point scale; all 21 items achieved an item-level content validity index (I-CVI) of 1.00 (scale-level content validity index based on the averaging method [S-CVI/Ave]=1.00). Evaluators also responded to a 3-option usage intent item (“usable as-is,” “usable with improvements,” and “currently unusable”). The complete rubric, anchored descriptors, and item-level analysis are reported in the companion paper [29].

### Coexistence Operating Modes

Three operating modes, all built on the same shared scenario base, NURSE-ONE × PATIENT-ONE pair, and rendering pipeline (Figure 3), are described below:

- Mode A—AI-AI demonstration: both slots were filled by AI; the learner observed a complete simulated encounter. The pedagogical purpose was exemplar observation, model dialogue exposure, and comparative analysis across scenarios.
- Mode B—student-as-nurse: the nurse slot was filled by the learner, and the patient slot was filled by PATIENT-ONE. NURSE-ONE was held in reserve as either B1 (reference mode)—producing a parallel “shadow” reply per turn for learner consultation—or B2 (feedback mode)—producing post hoc structured commentary on the learner’s utterance sequence. The pedagogical purpose was deliberate practice with structured scaffolding.
- Mode C—student-as-observer: mode A dialogues were rendered with an annotation overlay enabling learner labeling against the Berelson schema or a custom rubric. The pedagogical purpose was reflective evaluation and content analysis literacy.

A working web user interface (UI) implements all 3 modes (Multimedia Appendix 3). The same scenario set, character images, and voice synthesis pipeline (VOICEVOX/AivisSpeech) were reused across modes; only the agent-to-slot mapping differed.

**Figure 3.**
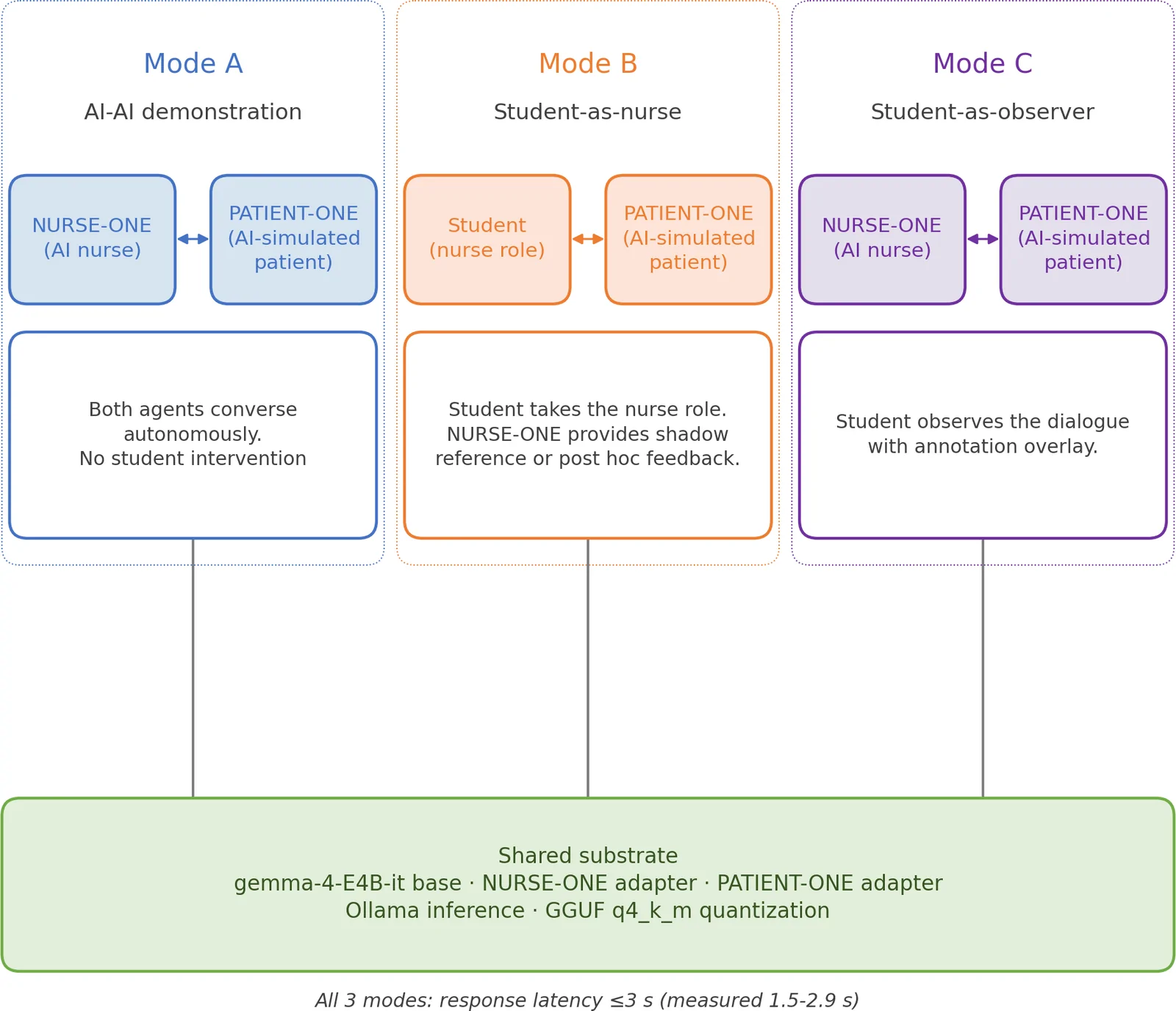
The 3 coexistence operating modes are built on the same shared substrate. In mode A, both agents converse as an AI-AI demonstration. In mode B, the student takes the nurse role, with NURSE-ONE providing either a shadow reference or post hoc feedback. In mode C, the student observes the dialogue with an annotation overlay.

### Operating Mode Feasibility Measurement

The UI was instrumented to record end-to-end response latency per agent turn (text generation only; voice synthesis was measured separately). The prespecified acceptance threshold was a median latency of ≤3 seconds per turn. For each mode, we documented the agent-to-slot mapping, the human input channel, and modal-specific artifacts (NURSE-ONE shadow output in B1; post hoc feedback in B2).

### Ethical Considerations

The study protocol was reviewed and approved by the Research Ethics Committee of Seirei Christopher University (approval 25-100-01; approved March 30, 2026). No human subject data were collected in the dialogue experiments: all dialogues reported here were AI-AI interactions, and analyses involved no identifiable human subject information. The third-party evaluation involved nursing faculty who provided informed consent; their responses are reported in aggregate with no individual identification. Dataset licensing was verified at first use; use of Japanese standardized patient training literature follows Japanese Copyright Act Article 30‐4 (use of works for information analysis).

The use of AI tools is disclosed in four categories: (1) Claude Code (Anthropic) as a development support tool for system implementation; (2) Claude Sonnet 4.5 and Claude Opus 4.6 as automated content analysis raters for Berelson classification, the methodological implications of which are examined in the *Discussion* section; (3) NotebookLM (Google) for preliminary figure drafting (all figures were subsequently redesigned for journal publication); and (4) AI assistance for manuscript language editing only (research design, analysis, and interpretation were carried out by the authors).

## Results

### Human Coder Interrater Reliability

Two nursing experts independently classified 196 blinded utterances (4 missing responses excluded from the original 200) using the 6-category Berelson scheme. Overall Cohen κ was 0.770 (82.7% agreement), representing substantial agreement [28]. Condition-level κ values ranged from 0.645 (base condition) to 0.839 (prototype nurse 2, 735-case condition), indicating that classification reliability was maintained across all 4 conditions and was not driven by any single condition or scenario cluster (Table 1). Category-level agreement rates ranged from 57.6% (CQ) to 85.7% (IN), with the highest agreement on categories with clear behavioral anchors (IN and AC) and lower agreement on categories requiring more inferential judgment (CQ, EM, and OT) (Multimedia Appendix 4).

For comparison, the 2 LLM raters (Claude Sonnet 4.5 and Claude Opus 4.6) achieved κ=0.742 on a separate subsample (n=100, seed=42). The difference between human interrater κ (0.770) and LLM interrater κ (0.742) was 0.028; both values fall within the “substantial agreement” band [28]. The comparability of these values supports the use of LLM-assisted classification for the full-dataset analysis, a point examined further in the *Discussion* section.

**Table 1.** Human coder interrater reliability by condition.

Condition	Set	κ	Agreement (%)	Utterances, n
Base (gemma4-E4B-it)	A	0.645	73.5	49
Prototype nurse 2 (735 cases)	B	0.839	87.8	49
Prototype nurse 1 (245 cases)	C	0.778	83.7	49
Prototype nurse 3 (1225 cases)	D	0.809	85.7	49
Overall	—^a^	0.770	82.7	196

a Not applicable.

### Four-Condition Berelson Analysis: Nurse-Side Training Volume Effects

The 4-condition omnibus comparison yielded *χ*^2^_15_=42.0 (*P*<.001; significant).

The OQ rate reached its maximum at prototype nurse 2 (preset × 3, 735 cases; OQ=21.5%) and decreased at prototype nurse 3 (preset × 5, 1225 cases; OQ=15%). The IN rate, in contrast, was maximized at prototype nurse 3 (IN=15.5%, approximately 4× the gemma4-E4B-it base rate of ~3.5%). Thus, the relationship between training data volume and Berelson indicators was nonmonotonic: OQ optimization favored 735 cases, while IN maximization favored 1225 cases (Figure 4). On the basis of OQ as the primary criterion for partner data quality in the bidirectional pipeline—a rationale examined in the *Discussion* section—prototype nurse 2 was adopted as NURSE-ONE and selected as the data collection partner for PATIENT-ONE training. The final loss values decreased monotonically with volume (2.36 → 1.537 → 0.840) but did not covary with OQ; loss reduction is therefore a necessary but not sufficient indicator of dialogic quality.

**Figure 4.**
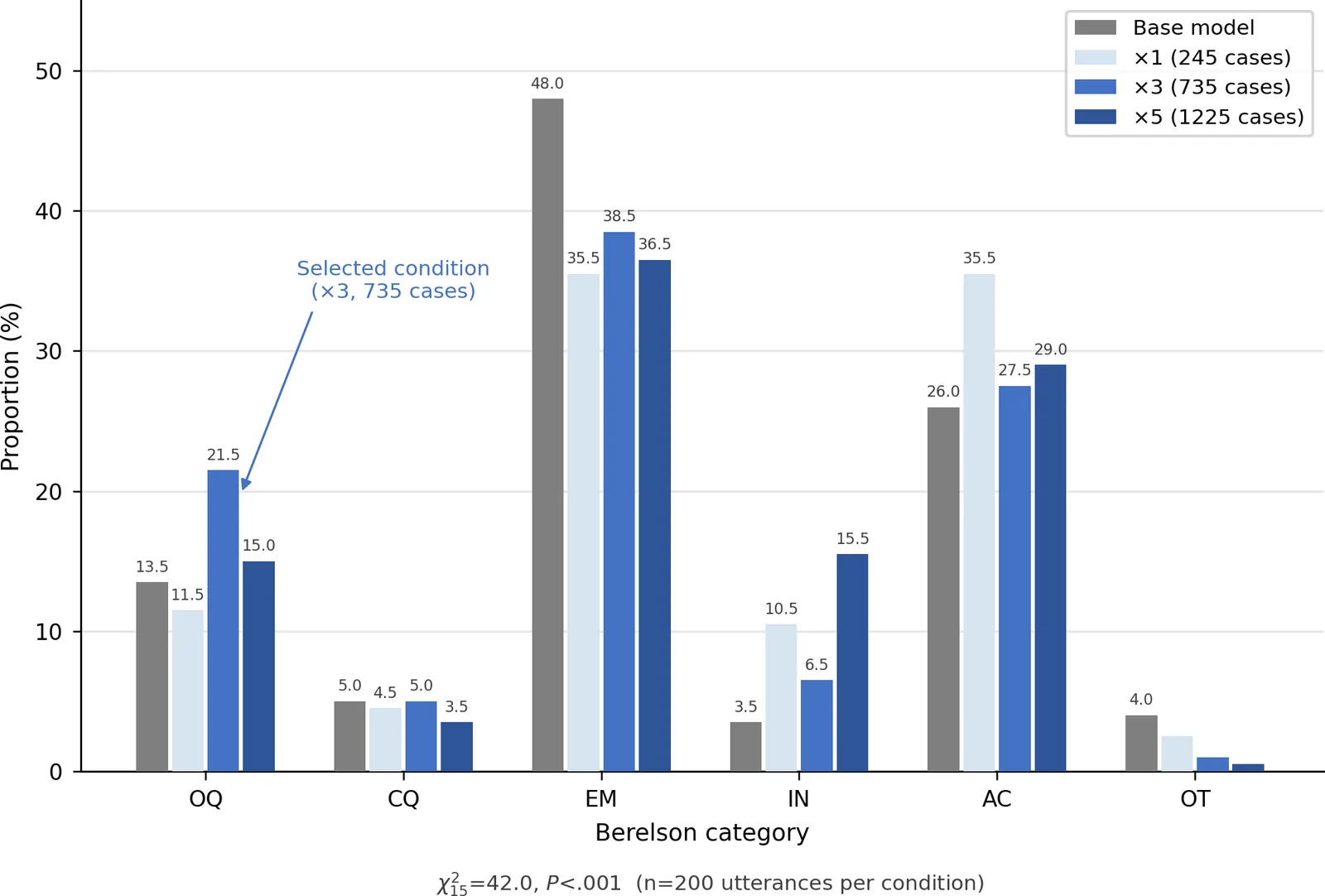
Nurse-side Berelson category distribution across 4 training volume conditions (n=200 utterances per condition). OQ peaks at ×3/735 cases (21.5%); IN peaks at ×5/1225 cases (15.5%). The nonmonotonic relationship between training volume and OQ indicates that loss reduction alone is not a sufficient indicator of dialogic quality. AC: assessment confirmation; CQ: closed question; EM: empathic acknowledgment; IN: information collection; OQ: open question; OT: other.

### PATIENT-ONE A/B/C Berelson Analysis

Interrater reliability for patient-side classification was κ=0.718 on a C-condition sample (n=100, seed=42), satisfying the adoption threshold. The 3-condition omnibus comparison yielded *χ*^2^_10_=11.8 (*P*=.30; not significant).

Categorically, patient-side fine-tuning did not shift the Berelson distribution—a striking contrast with the nurse-side significant effect. However, quantitatively, both fine-tuned conditions (B and C) reduced response length by approximately 33% relative to base (Table 2); condition C achieved the lowest within-session response length variability (SD 24.5 characters per utterance) and the highest patient-side IN (information-providing utterances, 47.9%), with AC=4.2% (identical to base AC=4.2%). Prototype patient 2 (condition C) was adopted as PATIENT-ONE. Condition B (prototype patient 1, preset × 1) showed an isolated AC excess (z=+2.30, *P*=.02).

**Table 2.** Patient-side A/B/C comparison (n=240 utterances per condition).

Condition	Training cases	Response length (characters), mean (SD)	AC^a^ (%)	CQ^b^ (%)	EM^c^ (%)	IN^d^ (%)	OQ^e^/OT^f^ (%)
A (base)	0	76.1 (29.8)	4.2	0.4	32.5	43.3	5.0/14.6
B (prototype patient 1)	245	51.1 (27.2)	8.3	1.7	26.2	40.4	5.4/17.9
C (prototype patient 2, adopted as PATIENT-ONE)	735	51.1 (24.5)	4.2	1.2	27.9	47.9	3.8/15.0

a AC: assessment confirmation.

b CQ: closed question.

c EM: empathic acknowledgment.

d IN: information collection.

e OQ: open question.

f OT: other.

The selection of condition C over condition B was exploratory rather than prespecified: no threshold for response length variability or IN rate was defined in advance of observing the results for the 3 conditions. A post hoc comparison (Multimedia Appendix 5) confirmed that mean response length was statistically indistinguishable between conditions B and C (Welch *t*=0.004, *P*>.99; Cohen *d*=0.000), and Levene test found no significant difference in variability between conditions B and C (*P*=.14) or across all 3 conditions (*P*=.12). The strongest and only statistically robust effect was the length reduction from A to B or C (both *P*<.001; Cohen *d*=0.87‐0.92), reflecting the impact of fine-tuning per se rather than a clear advantage of C over B. Condition C was ultimately adopted on the basis of its marginally higher IN rate (47.9% vs 40.4%) and marginally lower SD for response length (24.5 vs 27.2), consistent with the study’s descriptive rather than confirmatory framing of the A/B/C comparison.

### Functional Asymmetry Between Nurse-Side and Patient-Side Fine-Tuning

Comparing the 2 experiments (Table 3) revealed that the nurse-side adapter and the patient-side adapter operated on different dimensions of dialogic behavior. Nurse-side training shifted the qualitative composition of utterance categories (the nurse asked differently), with significant *χ*^2^ values and direct OQ or IN shift. Patient-side training shifted the quantitative profile of utterance length (the patient was more concise and consistent), with no significant category shift. Neither adapter produced effects on the other’s principal axis. We refer to this as a functional asymmetry and present it, on the basis of a single pipeline and a single base model, as a preliminary observed pattern rather than an established structural property; replication across additional pipelines and base models is needed before it can be treated as more than an incidental observation.

**Table 3.** Functional asymmetry between nurse-side and patient-side fine-tuning.

Property	Nurse-side adapter (NURSE-ONE)	Patient-side adapter (PATIENT-ONE)
Sample size, n	200 utterances × 4 conditions	240 utterances × 3 conditions
Interrater reliability (κ)	0.742 (LLM^a^); 0.770 (human)	0.718 (LLM)
Berelson omnibus statistic		
Chi-square (*df*)	42.0 (15)	11.8 (10)
*P* value	<.001	.30
Effect on category distribution	Significant qualitative shift (eg, OQ^b^ 13.5% → 21.5%)	Not significant (no category distribution shift)
Effect on response length	Not the primary axis	Approximately 33% reduction; lowest SD 24.5
Operating axis	Qualitative (questioning style)	Quantitative (response stability)

a LLM: large language model.

b OQ: open question.

**Table 4.** Third-party evaluation results by subscale (n=10 nursing faculty).

Subscale	Target	Items, n	Score (mean/max)	Achievement (%)	Cronbach α
A	AI nurse	8	27.6/40	69.0	.792
B	AI-simulated patient	8	36.1/40	90.3	.718
C	Dyadic interaction	5	21.2/25	84.8	.799

### Third-Party Expert Evaluation

Ten nursing faculty members evaluated the NURSE-ONE × PATIENT-ONE system using the 21-item rubric. Table 4 summarizes the subscale-level results.

Subscale B (AI-simulated patient) achieved the highest rating at 90.3%. Two items reached ceiling scores across all 10 evaluators (mean 5, SD 0): patient identity consistency and conversational responsiveness. The subscale A (AI nurse) was rated at 69%, with the highest item score on empathic listening (mean 3.9) and the lowest on clinical judgment and nursing intervention (both means 3). The subscale C (dyadic interaction) was rated at 84.8%, with role maintenance achieving a ceiling score (mean 5, SD 0) across all evaluators—consistent with the finding of zero role collapse in the coexistence interaction analysis.

Regarding usage intent, 2 of 10 (20%) evaluators rated the system as “usable as-is,” 8 of 10 (80%) as “usable with improvements,” and 0 of 10 (0%) as “currently unusable.” Free-text comments identified insufficient depth of nursing intervention (7 evaluators) and limited exploration of psychosocial background (6 evaluators) as primary improvement targets—areas corresponding to the subscale A items with the lowest scores (clinical judgment and nursing intervention). Positive evaluations centered on empathic responsiveness and the system’s suitability for repeated practice.

### Coexistence Operating Modes—Feasibility

A working web UI instantiated all 3 modes across the 20-scenario subset used for interaction analysis. Median per-turn latency remained within the prespecified ≤3-second threshold across all modes.

Mode A (AI-AI demonstration) generated complete encounters end to end without human intervention. Scenarios were completed within the maxmsg=25 budget in the majority of runs; truncation, when it occurred, was driven by clinical end point and not by computational limit.

Mode B (student-as-nurse) bound the nurse slot to the human input channel; PATIENT-ONE remained as the dialogic partner. B1 (reference) displayed NURSE-ONE’s shadow reply alongside the learner’s utterance as a nonintrusive reference. B2 (feedback) withheld NURSE-ONE during the encounter and prompted it at the end to produce structured commentary on the learner’s utterance sequence.

Mode C (student-as-observer) rendered mode A dialogues with an annotation overlay for the Berelson schema or custom rubrics. Multiple observers can rate the same dialogue, supporting cross-observer comparison and instructor-led discussion.

All 3 modes shared the same scenario set, agent pair, and rendering pipeline; only the agent-to-slot mapping differed.

### Coexistence Interaction: Nurse-Side Effects on Patient-Side Behavior

A complementary interaction analysis on a 20-scenario × 2-run subset (mode A) compared 2 nurse conditions—base × PATIENT-ONE vs NURSE-ONE × PATIENT-ONE—holding the patient role constant. Patient response length showed scenario-dependent variation under a nurse condition (cross-scenario mean +12.7%, SD 48.8, range −59.3% to +99.9%; direction mixed across scenarios with no statistically reliable overall increase). Larger amplifications were observed in scenarios privileging open-ended assessment (eg, heart failure, hypertensive pregnancy, depression, parkinsonism, early dementia, and disaster triage); larger contractions were observed in scenarios favoring focused or affect-heavy patient narratives (eg, normal pregnancy, amyotrophic lateral sclerosis [ALS], schizophrenia, and end-of-life). Patient-side category distribution and lexical diversity did not differ significantly between nurse conditions, consistent with the patient-side category stability finding reported above. Role collapse was zero across all dialogues. These observations are consistent with—rather than a direct test of—the functional asymmetry: under coexistence, the patient-side category stability is preserved, while patient response length varies in a scenario-dependent manner that is itself in keeping with nurse-side qualitative axis modulation. A confirmatory test with broader scenario coverage and additional nurse condition contrasts is identified as future work.

## Discussion

### Principal Findings

We constructed 2 specialized fine-tuned LLMs—NURSE-ONE and PATIENT-ONE—via a 5-step bidirectional pipeline and showed that their joint deployment supported a coherent dyadic simulation system. Four findings are central. First, the relationship between nurse-side training data volume and Berelson dialogic indicators was nonmonotonic: 735 cases optimized OQ (21.5%), and 1225 cases maximized IN (15.5%). Second, patient-side fine-tuning produced no significant Berelson category shift but reliably reduced response length variability and shortened utterances by ~33%. Third, the 2 adapters appeared to operate on different axes—qualitative on the nurse side and quantitative on the patient side—a preliminary observed functional asymmetry that survived coexistence and appeared to underwrite 3 pedagogically distinct operating modes within a ≤3-second latency envelope. Fourth, third-party evaluation by 10 nursing faculty confirmed exploratory educational applicability, with the AI-simulated patient rated at 90.3%, and 100% (10/10) of evaluators rating the system as usable.

### Resolution of the Prior Local Deployment Limitations

The authors’ prior work [29] identified 3 obstacles to the local deployment of digital simulated patient systems: response speed, specialty insufficiency, and difficulty in maintaining patient individuality. The 5-step bidirectional pipeline addresses all 3.

- Response speed: gemma4-E4B-it × gemma4-E4B-it dyadic exchange at 2.5 to 2.9 seconds per turn satisfies the ≤3-second educational threshold without cloud inference.
- Specialty insufficiency: nurse-side fine-tuning lifts OQ from ~13.5% (base) to 21.5% (NURSE-ONE) and IN from ~3.5% to 15.5% under the IN-priority condition.
- Patient individuality: the 3-layer architecture localizes individuality in layer 3 (scenario prompt), allowing a single PATIENT-ONE LoRA to be combined with all 251 scenarios.

### Effectiveness of the Bidirectional Fine-Tuning Approach

Generating PATIENT-ONE training data from a fine-tuned nurse partner rather than an unspecialized one is the central structural choice of this study. The patient-side A/B/C result (no significant category shift but ~33% length reduction and SD minimization) is consistent with high data quality from the fine-tuned partner: when the partner’s questions are well formed (high OQ and balanced category distribution), the patient model trained on the resulting dialogues converges on stable, focused responses rather than degenerate or overlong outputs.

A potential concern with the bidirectional pipeline is epistemic circularity: because PATIENT-ONE is trained on NURSE-ONE–generated dialogues, the patient model might merely reflect the nurse model’s linguistic idiosyncrasies rather than learning generalizable patient response patterns. Three observations mitigate this concern. First, the functional asymmetry itself constitutes evidence against simple echo: if the patient model were merely reproducing nurse-side patterns, we would expect a significant shift in patient-side category distribution mirroring the nurse-side shift; yet, no such shift was observed (*χ*^2^_10_=11.8, *P*=.30). The patient-side adapter operated on a different axis (response length and stability) from the nurse-side adapter (category distribution), indicating that the 2 models learned distinct, complementary behavioral dimensions rather than a shared linguistic surface. Second, the third-party evaluation provided external human validation: 10 nursing faculty rated PATIENT-ONE at 90.3%, with patient identity consistency and conversational responsiveness achieving ceiling scores—assessments that would be unlikely if the patient model were producing nurse-like rather than patient-like responses. Third, role collapse was zero across all coexistence dialogues, confirming that the models maintained role-appropriate behavior under joint deployment. Nevertheless, we note that a direct comparison between patient models trained on fine-tuned nurse dialogues vs base-nurse dialogues—which the present study did not include—would provide stronger evidence for the bidirectional approach and is identified as future work.

We further note that this concern is structurally distinct from the model collapse phenomenon documented for iterative self-consuming training loops [32,33], in which a single model lineage is repeatedly retrained on its own prior synthetic outputs across many generations, progressively narrowing distributional diversity in the absence of fresh real data. The present pipeline differs on both counts: (1) NURSE-ONE’s own training corpus was entirely human-authored (institutional care plans and national examination items) and contained no synthetic data, and (2) the pipeline executed a single, 1-directional transfer—NURSE-ONE generated the training corpus for PATIENT-ONE once, and NURSE-ONE was never retrained on PATIENT-ONE’s outputs—rather than the multigeneration self-consumption loop that drives collapse in those studies [32,33]. This structure is closer to conventional cross-model knowledge distillation, in which a trained teacher model generates training signal for a structurally distinct student model, than to the self-consuming regime described in the model collapse literature. We present the pipeline as a technical feasibility demonstration rather than a claim of collapse-free scalability; extending it to additional generations or bidirectional retraining cycles was not evaluated in this study and would require the safeguards documented in that literature, such as retaining a persistent fraction of real data at each generation.

### Comparison With Prior Work

The 7 principal LLM-based simulated patient projects surveyed in the *Introduction* [9-15] focus exclusively on the patient side; none specialize the nurse role. Recent reviews [16,17] confirm the gap. The present work is, to our knowledge, the first to fine-tune both roles and to analyze their joint behavior empirically. Against benchmark accuracy–driven approaches such as Yu et al [13], the present Berelson methodology captures how the dialogue is conducted and not only whether the right facts are produced—a distinction central to nursing education where communication style is itself a learning target.

### Functional Asymmetry as a Candidate Design Principle

The nurse-side adapter modulated qualitative questioning style, and the patient-side adapter controlled quantitative response stability. This preliminarily observed functional asymmetry suggests a candidate design principle, pending replication across additional pipelines and base models: when fine-tuning 2 interacting roles, allocate qualitative variation to one role and quantitative stability to the other; do not optimize both axes within a single adapter. The 2 adapters then compose under coexistence rather than compete on a shared axis. Three corollary principles follow (Figure 5): shared substrate (layer 1 + layer 3) with role-specific adapter (layer 2), reuse of a single AI-AI dialogue substrate (mode A) as the basis for practice (mode B) and reflective evaluation (mode C) without per-mode authoring, and treating the dialogic partner as a first-class research target rather than as static infrastructure.

**Figure 5.**
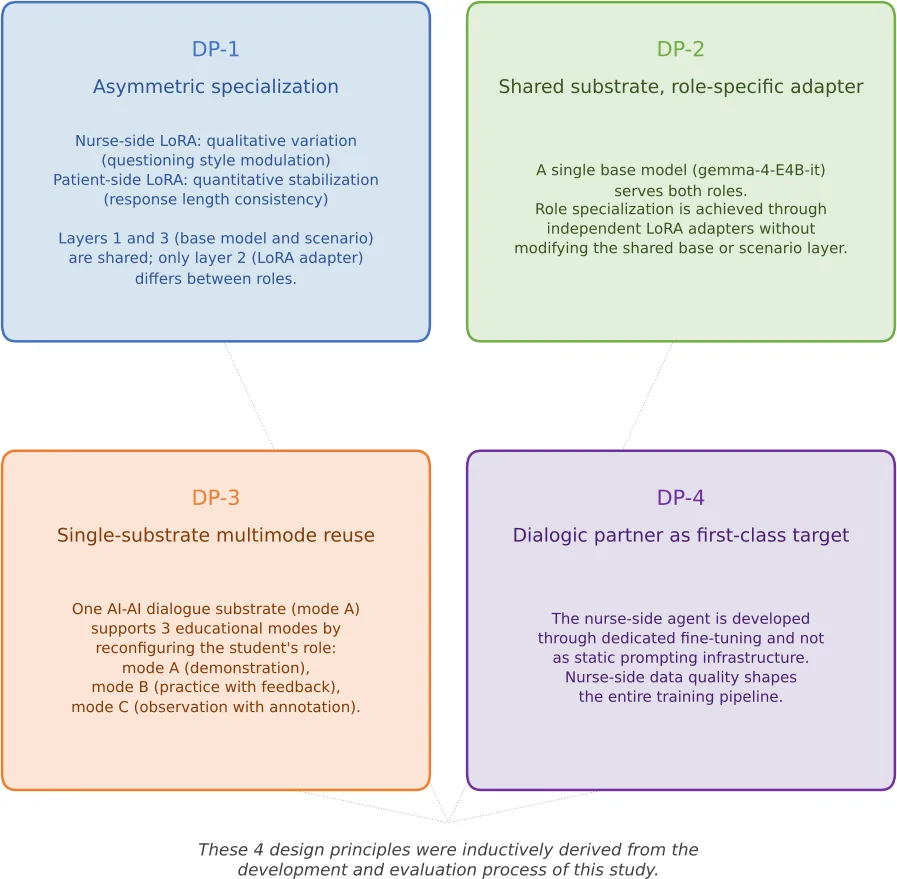
Four candidate design principles for AI-driven nursing simulation ecosystems derived from the preliminarily observed functional asymmetry. DP: design principle; LoRA: low-rank adaptation.

### Open Question vs Information Collection Trade-Off

The selection of the 735-case model (OQ=21.5%) over the 1225-case model (IN=15.5%) as the production NURSE-ONE warrants explicit justification, as both indicators represent valuable clinical competencies [25].

Open-ended questioning is widely recognized as a core nursing communication skill. The SEGUE framework for medical communication [30] emphasizes eliciting the patient’s perspective through open-ended prompts before transitioning to focused inquiry. In the bidirectional pipeline, the nurse model serves a dual function: it is both a pedagogical reference and the data collection partner whose dialogues become PATIENT-ONE’s training corpus. OQ-rich nurse utterances elicit longer, more detailed patient responses—providing richer training signal for patient-side fine-tuning—whereas IN-heavy utterances, while clinically efficient, tend to elicit brief confirmatory responses that offer less variation for patient model learning.

The trade-off, however, is not zero-sum in practice. The 1225-case model’s elevated IN (15.5%, ~4× baseline) demonstrates that deeper training drives the nurse model toward a more information gathering–style characteristic of experienced practitioners [26]. This finding is consistent with Benner’s observation [25] that expert nurses integrate assessment more seamlessly into conversation. The nonmonotonic relationship between training volume and OQ—rising at 735 cases then declining at 1225 cases—suggests that the OQ-to-IN shift may reflect a qualitative transition in dialogic strategy rather than mere degradation of questioning diversity. Future work should explore whether a curriculum that exposes learners to both the OQ-optimized (735-case) and IN-optimized (1225-case) nurse models could leverage this trade-off as a teaching tool for demonstrating the novice-to-expert communication trajectory.

### Third-Party Evaluation and Educational Applicability

The third-party evaluation by 10 nursing faculty provides exploratory evidence of educational applicability that the Berelson content analysis alone cannot establish. The asymmetry in subscale achievement rates—AI-simulated patient at 90.3% vs AI nurse at 69.0%—is consistent with the functional asymmetry at the model level: the patient-side LoRA, which stabilizes response patterns, produces behavior that experts rate more favorably, while the nurse-side LoRA, which diversifies questioning style, operates in a domain (clinical judgment and nursing intervention) where expert evaluators hold higher standards.

The gap between the nurse and patient subscales identifies specific improvement targets: clinical judgment (mean 3) and nursing intervention (mean 3) were the lowest-rated items, precisely the areas where fine-tuning is most constrained by the base model’s medical reasoning capacity rather than by dialogic pattern learning. The ceiling scores on role maintenance (subscale C) and patient identity consistency (subscale B) confirm that the 3-layer architecture successfully preserves role integrity across evaluators and scenarios.

Importantly, the evaluator sample (n=10) limits these findings to exploratory status. The results do not establish validated educational effectiveness; rather, they confirm that the system’s outputs are perceived as educationally relevant by domain experts and identify specific dimensions for iterative improvement. A learner outcome study with pre-post measurement is necessary to establish educational effectiveness.

### Validation of LLM-as-Rater Methodology

The present study used LLM-assisted classification as the primary analytical method for the full Berelson dataset, a methodological choice that warranted validation. The human coder validation (κ=0.770, substantial agreement) and the LLM interrater reliability (κ=0.742, substantial agreement) provide complementary evidence for the classification scheme’s robustness.

Three points support the validity of the LLM-assisted approach. First, both human and LLM κ values fall within the same interpretive band (substantial agreement, 0.61‐0.80), with a difference of only 0.028. This indicates that the LLM raters achieved a level of classification consistency comparable to that of trained human experts using the same category definitions. Second, the category-level agreement patterns are consistent: both human and LLM raters showed highest agreement on categories with clear behavioral anchors (IN and AC) and lower agreement on more inferential categories (CQ, EM, and OT), suggesting that the sources of classification difficulty are inherent to the category scheme rather than specific to rater type. Third, a potential concern with using 2 LLMs from the same provider (Anthropic) is shared architectural bias. However, Claude Sonnet 4.5 and Claude Opus 4.6 differ in model size, architecture, and training mix; the observed κ=0.742 is not notably higher than the human κ=0.770, providing no evidence for an inflated-agreement artifact.

We note that LLM-as-rater methodology does not replace human judgment but serves as a scalable complement. The present study demonstrates a practical validation pathway: validate the classification scheme with a human coder subsample, and then apply LLM-assisted classification to the full dataset when the scale of data (800+ utterances across multiple conditions) exceeds what is feasible for manual coding.

### Coexistence and Disaster Preparedness Applicability

A consequence of the local-only architecture (RTX 4090 + Ollama + on-device GGUF) is that the entire system continues to operate when cloud LLM APIs and conventional in-person standardized patient infrastructure are unavailable. This includes pandemic settings [6], armed conflict environments [4,5], and natural disaster contexts where communications and physical mobility are degraded.

The deployment requirements are concrete and modest (Figure 6): the complete system—2 merged GGUF models (5.07 GB each), Ollama runtime, web UI, and voice synthesis engine—occupies approximately 15 GB of storage and runs on any NVIDIA GPU with ≥16 GB VRAM (Turing architecture or later). A laptop-class mobile workstation with a portable uninterruptible power supply constitutes a self-contained simulation unit deployable to evacuation shelters, field hospitals, or university buildings operating on generator power. The 251-scenario base already includes a disaster item (large earthquake casualty triage) and an emergency or typhoon blackout ICU item from the national examination; the same mode A/B/C operating mode space applies to disaster preparedness simulation without modification.

We distinguish between technical deployment capability—demonstrated in this study—and validated deployment effectiveness in actual disaster preparedness settings, which remains future work. The former establishes that no external dependency (cloud API, internet connectivity, and standardized patient actor availability) gates the system’s operation; the latter requires field trials under realistic resource-constraint conditions.

**Figure 6.**
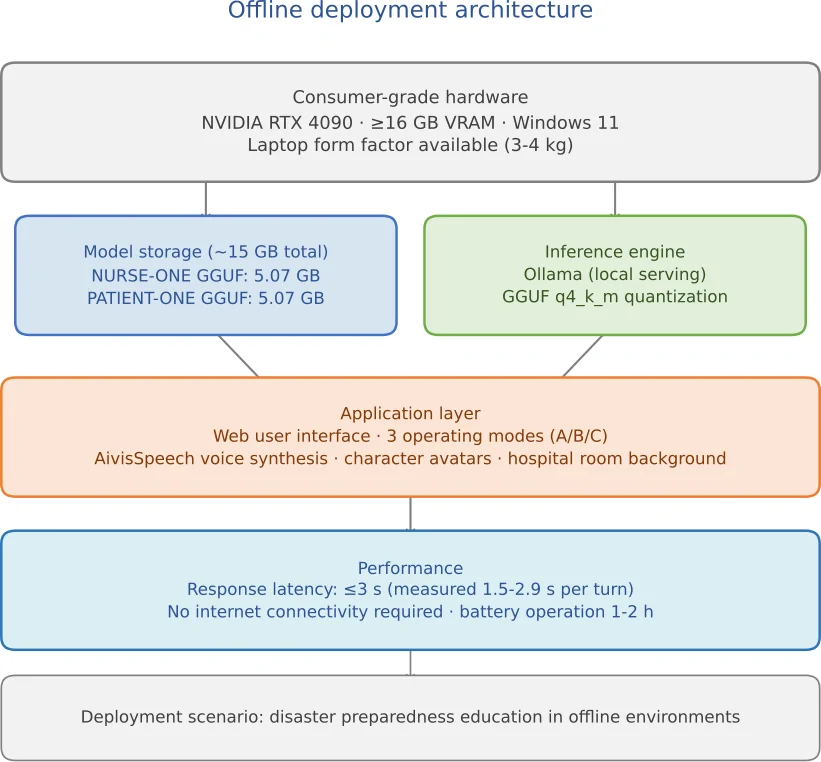
Offline deployment capability. The dual-agent ecosystem operates entirely on a single consumer graphics processing unit with merged GGUF models served via Ollama. Total storage ~15 GB; minimum video random access memory (VRAM) ≥16 GB (Turing architecture or later). A laptop-class workstation with portable uninterruptible power supply constitutes a deployable simulation unit.

### Limitations

The study has the following limitations:

1. The study was conducted in a single institutional context, with a single curriculum and in Japanese only.
2. A single base model (gemma4-E4B-it) was used, and whether the functional asymmetry persists with other base models remains open.
3. No learner outcome data were included; the operating modes were operationally instantiated and validated by expert evaluation, but knowledge, skill, and transfer outcomes are not yet measured.
4. Berelson’s 6-category schema captures dialogic surface structure well but cannot represent fine-grained clinical reasoning or clinical accuracy; the models’ outputs were not assessed for medical factual correctness.
5. PATIENT-ONE’s training corpus consists of NURSE-ONE–generated dialogues, inheriting any latent biases of the nurse-side adapter; a comparison between patient models trained on fine-tuned nurse dialogues vs base nurse dialogues was not conducted.
6. Coexistence mode analyses use a 20-scenario subset and a single nurse-condition contrast; broader scenario coverage and additional contrasts are needed.
7. The third-party evaluation sample (n=10) limits the findings to exploratory status; larger-scale evaluation with diverse institutional contexts is needed.
8. Individual utterances were treated as the primary unit of analysis; although scenario-level aggregation confirmed consistent patterns, multilevel modeling accounting for the nested structure of utterances within dialogues would provide more rigorous statistical inference.

### Future Work

Four directions follow: (1) a learner outcome study using modes B and C with first- and third-year nursing students, incorporating pre-post measurement of clinical communication skills; (2) a controlled comparison of PATIENT-ONE trained on fine-tuned nurse dialogues vs base nurse dialogues, directly testing the bidirectional pipeline’s added value; (3) multimodal integration with the authors’ existing 3D embodied agent pipelines (UE5 MetaHumans; VRoid [Pixiv Inc] VRM avatars for browser deployment) to add visual and auditory channels to the text-based simulation; and (4) cross-domain generalization to physician-patient and pharmacist-patient dyads to test the asymmetric specialization principle beyond nursing. A parallel disaster preparedness deployment study—integrating with existing earthquake, pandemic, and armed conflict educational material developed by the authors—will examine real-world offline operation under actual resource constraint conditions.

### Conclusions

A 5-step bidirectional fine-tuning pipeline produced 2 specialized models, NURSE-ONE and PATIENT-ONE, on a single consumer-grade GPU with a multimodality-preserving merge. Human coder validation confirmed the reliability of the Berelson classification scheme (κ=0.770, substantial agreement), and LLM-assisted classification (κ=0.742) produced comparable results, supporting the scalable analytical approach. Four-condition Berelson analysis (*χ*^2^_15_=42.0, *P*<.001) identified the 735-case nurse-side condition as the production NURSE-ONE on the basis of OQ optimization (21.5%), and the A/B/C analysis identified the 735-case patient-side condition as the production PATIENT-ONE on the basis of IN maximization (47.9%) and response length stabilization (SD 24.5). The 2 adapters appeared to operate on different axes—qualitative on the nurse side and quantitative on the patient side—a preliminary observed functional asymmetry that survived joint deployment and appears to underwrite 3 pedagogically distinct coexistence operating modes (AI-AI demonstration, student-as-nurse with reference or feedback scaffolding, and student-as-observer) within a ≤3-second latency envelope. Third-party evaluation by 10 nursing faculty confirmed exploratory educational applicability (AI-simulated patient: 90.3%; AI nurse: 69.0%; 100% [10/10] evaluators rated the system as usable). The system runs entirely offline on consumer-grade hardware, supporting nursing education simulation in disaster preparedness contexts where cloud-dependent or in-person actor–dependent alternatives are unusable. The technical feasibility and expert-validated applicability demonstrated here provide a foundation for future learner outcome studies. To our knowledge, this is the first reported coexisting dual fine-tuned LLM system for nursing education.

## Supplementary material

10.2196/100778 Multimedia Appendix 1 Extended description of the 5-step bidirectional fine-tuning pipeline and low-rank adaptation (LoRA) configuration.

10.2196/100778 Multimedia Appendix 2 Scenario base breakdown: 251 scenarios across 29 standard nursing care plans and 7 clinical domains.

10.2196/100778 Multimedia Appendix 3 User interface screenshots demonstrating the 3 coexistence operating modes in the web application.

10.2196/100778 Multimedia Appendix 4 Human coder validation data for Berelson content analysis.

10.2196/100778 Multimedia Appendix 5 Patient-side A/B/C statistical comparison (response length and category distribution).

10.2196/100778 Multimedia Appendix 6 Sample dialogues illustrating NURSE-ONE and PATIENT-ONE coexistence across 3 operating modes.

## Abbreviations

AC: assessment confirmation
ALS: amyotrophic lateral sclerosis
CQ: closed question
EM: empathic acknowledgment
GPU: graphics processing unit
I-CVI: item-level content validity index
IN: information collection
LLM: large language model
LoRA: low-rank adaptation
OP/TP/EP: observation plan, treatment plan, and education plan
OQ: open question
OT: other
PEFT: parameter-efficient fine-tuning
RQ: research question
S-CVI/Ave: scale-level content validity index based on the averaging method
SEGUE: set the stage, elicit information, give information, understand the patient’s perspective, and end the encounter
UI: user interface
VRAM: video random access memory

## References

[1] Cant RP, Cooper SJ (2010). Simulation-based learning in nurse education: systematic review. J Adv Nurs.

[2] Kim J, Park JH, Shin S (2016). Effectiveness of simulation-based nursing education depending on fidelity: a meta-analysis. BMC Med Educ.

[3] Lewis KL, Bohnert CA, Gammon WL (2017). The Association of Standardized Patient Educators (ASPE) Standards of Best Practice (SOBP). Adv Simul.

[4] Kagan I, Cohen O (2025). Enhancing resilience of nursing education during war: policy implications from a qualitative study. Isr J Health Policy Res.

[5] Mayer A, Yaremko O, Shchudrova T, Korotun O, Dospil K, Hege I (2023). Medical education in times of war: a mixed-methods needs analysis at Ukrainian medical schools. BMC Med Educ.

[6] Fogg N, Wilson C, Trinka M (2020). Transitioning from direct care to virtual clinical experiences during the COVID-19 pandemic. J Prof Nurs.

[7] Nakamura M, Yamasaki A, Watanabe S (2016). Current status and challenges of standardized patient training and utilization in Japanese nursing universities [Article in Japanese]. Bull Jpn Red Cross Hiroshima Coll Nurs.

[8] Singhal K, Azizi S, Tu T (2023). Large language models encode clinical knowledge. Nature.

[9] Du Z, Zheng L, Hu R (2025). Proceedings of the 63rd Annual Meeting of the Association for Computational Linguistics (Volume 1: Long Papers).

[10] Schmidgall S, Ziaei R, Harris C, Reis E, Jopling J, Moor M (2025). AgentClinic: a multimodal agent benchmark to evaluate AI in simulated clinical environments. arXiv.

[11] Kyung D, Chung H, Bae S (2026). Advances in Neural Information Processing Systems 38.

[12] Jiang S, Zhang Z, Zhu K, Bai T, Zhao R (2026). Multi-stage patient role-playing framework for realistic clinical interactions. arXiv.

[13] Yu H, Zhou J, Li L (2025). Simulated patient systems powered by large language model-based AI agents offer potential for transforming medical education. Commun Med (Lond).

[14] Li Y, Li Z, Zhang K, Dan R, Jiang S, Zhang Y (2023). ChatDoctor: a medical chat model fine-tuned on a Large Language Model Meta-AI (LLaMA) using medical domain knowledge. Cureus.

[15] Wei C, Chen Z, Zhang J, Liu J (2025). CCF International Conference on Natural Language Processing and Chinese Computing.

[16] Jo YW, Lee M, Yang HJ (2025). Large language model-based virtual patient simulations in medical and nursing education: a review. Applied Sciences.

[17] Li D, Lebai Lutfi S (2026). Large language model-based virtual patient systems for history-taking in medical education: comprehensive systematic review. JMIR Med Inform.

[18] Tu T, Schaekermann M, Palepu A (2025). Towards conversational diagnostic artificial intelligence. Nature.

[19] Fareez F, Parikh T, Wavell C (2022). A dataset of simulated patient-physician medical interviews with powerful AI tutors. Sci Data.

[20] Kononowicz AA, Woodham LA, Edelbring S (2019). Virtual patient simulations in health professions education: systematic review and meta-analysis by the digital health education collaboration. J Med Internet Res.

[21] Wang Y, Donovan H, Hassan S, Alikhani M (2023). MedNgage: a dataset for understanding engagement in patient-nurse conversations. Association for Computational Linguistics.

[22] Taira K, Yada S, Itaya T, Ogata S, Kiyoshige E, Hanada A (2026). Trilingual (EN/ZH-CN/JP) synthetic dataset of cerebral infarction patient-nurse bedside dialogs with metadata. medRxiv.

[23] Holtzman A, Buys J, Du L, Forbes M, Choi Y (2020). The curious case of neural text degeneration. arXiv.

[24] Li J, Galley M, Brockett C, Gao J, Dolan B Proceedings of the 2016 Conference of the North American Chapter of the Association for Computational Linguistics.

[25] Benner P (1984). From Novice to Expert: Excellence and Power in Clinical Nursing Practice.

[26] Sayar S, Tahmasebi R, Azodi P, Tamimi T, Jahanpour F (2018). The impact of tacit knowledge transfer through storytelling on nurses' clinical decision making. Iran Red Crescent Med J.

[27] Berelson B (1952). Content Analysis in Communication Research.

[28] Landis JR, Koch GG (1977). The measurement of observer agreement for categorical data. Biometrics.

[29] Koike T, Majima Y Development and evaluation of the simulated PATIENT LLM “PATIENT-ONE” using dialogue data with an AI nurse. Journal of Nursing Science and Engineering (forthcoming).

[30] Makoul G (2001). Essential elements of communication in medical encounters: the Kalamazoo consensus statement. Acad Med.

[31] Wind LA, Van Dalen J, Muijtjens AMM, Rethans JJ (2004). Assessing simulated patients in an educational setting: the MaSP (Maastricht Assessment of Simulated Patients). Med Educ.

[32] Shumailov I, Shumaylov Z, Zhao Y, Papernot N, Anderson R, Gal Y (2024). AI models collapse when trained on recursively generated data. Nature.

[33] Casco-Rodriguez J, Alemohammad S, Luzi L (2023). NIPS ’23: Proceedings of the 37th International Conference on Neural Information Processing Systems.

