# Supplementary material for: Coexistence of AI Nurse and AI-Simulated Patient in Nursing Education Using a Bidirectional Fine-Tuning Pipeline: Development and Content Analysis Study
Source: JMIR Biomed Eng. 2026 Nov 6;11:e100778. doi: 10.2196/100778 (PMC13743070; doi:10.2196/100778)
Supplement: Supplementary file 1 [file biomedeng-v11-e100778-s001.docx]

Multimedia Appendix 1

# The Five-Step Bidirectional Fine-Tuning Pipeline: Extended Description, Parameters, and Intermediate Artifacts

## Overview

This appendix provides the extended technical specification for the five-step bidirectional fine-tuning pipeline described in the Study Design and Overview section of the main text. Each step is documented with input/output artifacts, parameter values, and intermediate quality checks.

## Step 1: Scenario Base Construction

**Objective:** Assemble a comprehensive scenario base from which Layer-3 prompts and dialogue-collection runs are derived.

**Input:**

- 29 standard nursing care plans (authored by first author as HTML educational materials)
- 20 situational items from the 115th Japanese National Nursing Examination (February 2026)

**Process:**

- Each of the 29 nursing care plans was structured as a dialogue-ready scenario using OP/TP/EP (Observation / Treatment / Education) three-axis structure across seven domains: acute, chronic, geriatric, pediatric, psychiatric, maternal, and home-care.
- Each National Examination item was reconstructed as a simulated-patient scenario with patient profile (age, sex, diagnosis, hospitalization context), clinical scene, and correct nursing actions encoded as response policy in the nurse-side prompt.
- Each scenario was encoded into two paired prompts: (a) a nurse-side system prompt specifying the nurse role, clinical setting, and assessment goals; (b) a patient-side system prompt specifying the patient persona, chief complaints, affect, and speech register.

**Output:**

- **251-scenario base** (231 care-plan-derived + 20 examination-derived)
- Each scenario: nurse prompt + patient prompt + metadata (domain, disease, source type)

**Quality check:** Domain coverage validated against the Japanese national nursing curriculum standard (7 domains represented).

## Step 2: Provisional Dialogue System (AutoGen v18)

**Objective:** Collect multi-turn dialogues suitable for LoRA fine-tuning.

**Software:** Microsoft AutoGen (v18), configured for two-agent dialogue with per-scenario instantiation.

**Key parameters:**

| **Parameter** | **Value** | **Rationale** |
| --- | --- | --- |
| maxmsg | 25 | Calibrated via 400-trial experiment (§2.6.1) |
| Nurse model (initial) | gemma4-E4B-it (base) | Unspecialized baseline for Step 3 data |
| Nurse model (Step 5) | nurse-one (production) | Fine-tuned partner for patient data |
| Patient model | gemma4-E4B-it (base) | Unspecialized for Step 3; fine-tuned at Step 5 |
| Temperature | 0.7 (default) | Balanced diversity/coherence |
| Repetitions per scenario | ×1 / ×3 / ×5 | Three volume conditions for Step 3 |

**maxmsg calibration (the maxmsg Calibration section):**

| **Condition** | **Trials** | **seq-rep-4 max** | **Distinct-2 min** | **Degeneration rate** |
| --- | --- | --- | --- | --- |
| maxmsg = 20 | 100 | < 0.3 | > 0.5 | < 1% |
| maxmsg = 30 | 100 | < 0.3 | > 0.5 | < 1% |
| maxmsg = 40 | 100 | < 0.3 | > 0.5 | < 1% |
| maxmsg = 50 | 100 | < 0.3 | > 0.5 | < 1% |

**Output:**

- Step 3 corpus: 245 / 735 / 1225 multi-turn dialogues (×1 / ×3 / ×5 conditions)
- Step 5 corpus: dialogues generated by production NURSE-ONE × base patient

**Intermediate artifact:** JSONL files with OpenAI Chat-format messages (system / user / assistant roles), one line per dialogue session.

## Step 3: NURSE-ONE Fine-Tuning (Three Conditions)

**Objective:** Determine the effect of training-data volume on nurse-side dialogic behavior.

**Base model:** gemma4-E4B-it (multimodal, ~14.9 GB)

**Fine-tuning framework:** Unsloth + PEFT (LoRA)

**LoRA hyperparameters:**

| **Parameter** | **Value** |
| --- | --- |
| Rank (r) | 16 |
| Alpha | 32 |
| Target modules | q_proj, k_proj, v_proj, o_proj, gate_proj, up_proj, down_proj |
| Dropout | 0.05 |
| Learning rate | 2e-4 |
| Batch size | 4 (gradient accumulation: 4) |
| Epochs | 3 |
| Optimizer | AdamW (8-bit) |
| Max sequence length | 8192 |

**Three training-volume conditions:**

| **Condition** | **Label** | **Cases** | **Steps** | **Final loss** |
| --- | --- | --- | --- | --- |
| Prototype nurse 1 | preset × 1 | 245 | ~71 | 2.36 |
| Prototype nurse 2 | preset × 3 | 735 | ~142 | 1.537 |
| Prototype nurse 3 | preset × 5 | 1225 | ~308 | 0.840 |

**Output:**

- Three LoRA adapter checkpoints
- Training logs with per-step loss curves

## Step 4: Four-Condition Berelson Comparison (NURSE-ONE Selection)

**Objective:** Select the production NURSE-ONE from four conditions (base + 3 prototypes).

**Evaluation method:** Berelson content analysis with six-category schema (OQ, CQ, IN, AC, EM, OT)

**Raters:** Claude Sonnet 4.5 (primary) + Claude Opus 4.6 (secondary)

**Sample:** n = 200 utterances per condition (total 800 utterances)

**Inter-rater reliability:** κ = 0.742 (n=100, seed=42; two independent passes)

**Key results:**

| **Condition** | **OQ (%)** | **CQ (%)** | **IN (%)** | **AC (%)** | **EM (%)** | **OT (%)** |
| --- | --- | --- | --- | --- | --- | --- |
| Base (gemma4-E4B-it) | 13.5 | 54.0 | 3.5 | 9.0 | 10.0 | 10.0 |
| Prototype nurse 1 (×1) | 16.0 | 49.5 | 8.0 | 8.5 | 10.5 | 7.5 |
| Prototype nurse 2 (×3) | 21.5 | 45.0 | 10.5 | 7.0 | 9.0 | 7.0 |
| Prototype nurse 3 (×5) | 15.0 | 42.5 | 15.5 | 7.5 | 11.0 | 8.5 |

**Decision:** Prototype nurse 2 adopted as **NURSE-ONE** (OQ-maximizing criterion for partner-data quality).

**Omnibus statistic:** χ² = 41.991, df = 15, p = 0.0002

## Step 5: PATIENT-ONE Fine-Tuning and A/B/C Comparison

**Objective:** Fine-tune the patient role using NURSE-ONE-generated dialogue data; select production PATIENT-ONE.

**Data source:** Dialogues collected with NURSE-ONE (production) × gemma4-E4B-it (base patient)

**Two training-volume conditions:**

| **Condition** | **Label** | **Cases** | **Training data source** |
| --- | --- | --- | --- |
| Prototype patient 1 (B) | preset × 1 | 245 | NURSE-ONE × base patient |
| Prototype patient 2 (C) | preset × 3 | 735 | NURSE-ONE × base patient |

**Comparison conditions (A/B/C):**

- A: gemma4-E4B-it base patient (control)
- B: Prototype patient 1 (preset × 1)
- C: Prototype patient 2 (preset × 3)

**Sample:** n = 240 utterances per condition (total 720 utterances)

**Inter-rater reliability:** κ = 0.718 (C-condition n=100, seed=42)

**Omnibus statistic:** χ² = 11.818, df = 10, p = 0.297 (not significant)

**Key quantitative results:**

| **Condition** | **Mean response length (chars)** | **SD** | **IN (%)** |
| --- | --- | --- | --- |
| A (base) | ~42 | ~35 | 42.1 |
| B (×1) | ~28 | ~30 | 44.5 |
| C (×3) | ~27 | 24.5 | 47.9 |

**Decision:** Prototype patient 2 (condition C) adopted as **PATIENT-ONE** (lowest SD, highest IN).

## Model Merging and Deployment (Post-Pipeline)

**Merge method:** PEFT merge with `exclude_modules` preserving:

- `vision_tower` (vision processing)
- `audio_tower` (audio processing)
- `multi_modal_projector` (multimodal projection)

**GGUF conversion:** llama.cpp (b8793), q4_k_m quantization

**Final artifact sizes:**

| **Model** | **Merged checkpoint** | **GGUF q4_k_m** |
| --- | --- | --- |
| NURSE-ONE | ~14.92 GB | 5.07 GB |
| PATIENT-ONE | ~14.92 GB | 5.07 GB |

**Inference serving:** Ollama (local, no cloud dependency)

**Registered model names:** `nurse-one:v1.0`, `patient-one:v1.0`

## Intermediate Artifact Summary

| **Step** | **Artifact** | **Format** | **Count** |
| --- | --- | --- | --- |
| 1 | Scenario base | JSON (nurse prompt + patient prompt) | 251 |
| 2 | Dialogue corpus (nurse training) | JSONL (Chat format) | 245 / 735 / 1225 |
| 2 | Dialogue corpus (patient training) | JSONL (Chat format) | 245 / 735 |
| 3 | Nurse LoRA adapters | SafeTensors | 3 |
| 4 | Berelson classification results | CSV / JSON | 800 utterances |
| 5 | Patient LoRA adapters | SafeTensors | 2 |
| 5 | Berelson classification results | CSV / JSON | 720 utterances |
| Post | Merged model checkpoints | SafeTensors | 2 |
| Post | GGUF quantized models | GGUF q4_k_m | 2 (5.07 GB each) |
