# Supplementary material for: Coexistence of AI Nurse and AI-Simulated Patient in Nursing Education Using a Bidirectional Fine-Tuning Pipeline: Development and Content Analysis Study
Source: JMIR Biomed Eng. 2026 Nov 6;11:e100778. doi: 10.2196/100778 (PMC13743070; doi:10.2196/100778)
Supplement: Supplementary file 2 [file biomedeng-v11-e100778-s002.docx]

Multimedia Appendix 2

# 251-Scenario Base: Domain × Disease × Source-Type Breakdown

## Overview

The 251-scenario base serves as the shared input substrate for both Layer-3 prompts and dialogue-collection runs. It comprises two source types: (a) 231 scenarios derived from 29 institutionally-authored standard nursing care plans, and (b) 20 scenarios reconstructed from the 115th Japanese National Nursing Examination (administered February 2026).

## Source-Type Summary

| **Source type** | **Scenarios** | **Percentage** |
| --- | --- | --- |
| Standard nursing care plans (29 diseases × variants) | 231 | 92.0% |
| 115th National Nursing Examination (20 situational items) | 20 | 8.0% |
| Total | 251 | 100% |

## Domain × Disease Breakdown: Standard Nursing Care Plans (29 Diseases)

Each disease was authored as a standard nursing care plan with OP/TP/EP (Observation / Treatment / Education) three-axis structure. Multiple scenario variants (patient age, sex, family composition, disease stage, complication) were derived from each disease to reach the 231-scenario total.

| **#** | **Domain** | **Disease** | **Scenario variants** |
| --- | --- | --- | --- |
| 1 | Acute | Heart failure (acute exacerbation) | 8 |
| 2 | Acute | Brain hemorrhage / Subarachnoid hemorrhage | 8 |
| 3 | Acute | Pneumonia (community-acquired) | 8 |
| 4 | Acute | Lung cancer (perioperative) | 8 |
| 5 | Acute | Esophageal cancer (perioperative) | 8 |
| 6 | Acute | Colorectal cancer (perioperative) | 8 |
|  |  | Acute subtotal | 48 |
| 7 | Chronic | Type 2 diabetes mellitus | 8 |
| 8 | Chronic | COPD (chronic management) | 8 |
| 9 | Chronic | Chronic kidney disease / Hemodialysis | 8 |
| 10 | Chronic | Rheumatoid arthritis | 8 |
|  |  | Chronic subtotal | 32 |
| 11 | Geriatric | Alzheimer-type dementia | 8 |
| 12 | Geriatric | Parkinson's disease | 8 |
| 13 | Geriatric | Hip fracture (fall-related) | 8 |
| 14 | Geriatric | End-of-life care / Bereavement | 8 |
|  |  | Geriatric subtotal | 32 |
| 15 | Pediatric | Childhood leukemia (ALL) | 8 |
| 16 | Pediatric | Childhood asthma | 8 |
| 17 | Pediatric | Kawasaki disease | 8 |
|  |  | Pediatric subtotal | 24 |
| 18 | Psychiatric | Major depressive disorder | 8 |
| 19 | Psychiatric | Schizophrenia | 8 |
| 20 | Psychiatric | Bipolar disorder | 8 |
| 21 | Psychiatric | Eating disorder (anorexia nervosa) | 8 |
|  |  | Psychiatric subtotal | 32 |
| 22 | Maternal | Normal pregnancy / Childbirth | 8 |
| 23 | Maternal | Pregnancy-induced hypertension | 8 |
| 24 | Maternal | Preterm labor | 8 |
| 25 | Maternal | Postpartum depression | 7 |
|  |  | Maternal subtotal | 31 |
| 26 | Home-care | ALS (amyotrophic lateral sclerosis) | 8 |
| 27 | Home-care | COPD with home oxygen therapy | 8 |
| 28 | Home-care | Cerebrovascular disease (home rehabilitation) | 8 |
| 29 | Home-care | Cancer palliative care (home hospice) | 8 |
|  |  | Home-care subtotal | 32 |
|  |  | Care plan total | 231 |

## Domain × Disease Breakdown: 115th National Nursing Examination (20 Items)

| **EP#** | **Domain** | **Clinical scenario** | **Patient profile** |
| --- | --- | --- | --- |
| EP01 | Adult (rehabilitation) | Post-stroke home discharge | 80M, right hemiplegia, mild aphasia |
| EP02 | Adult (surgical) | Esophageal cancer postoperative | 65M, pain control, oral intake |
| EP03 | Adult (chronic) | Diabetic nephropathy CAPD | 58M, self-management education |
| EP04 | Geriatric | Lewy body dementia (early) | 75M, visual hallucinations, fall risk |
| EP05 | Pediatric | Childhood asthma (acute) | 10M, post-exercise wheeze |
| EP06 | Pediatric | Nephrotic syndrome (child) | 6M, edema, diet restrictions |
| EP07 | Pediatric | Cerebral palsy (developmental) | 5F, family support, rehabilitation |
| EP08 | Maternal | High-risk pregnancy (twin) | 32F, gestational diabetes |
| EP09 | Maternal | Postpartum hemorrhage | 30F, emergency intervention |
| EP10 | Emergency/Disaster | Earthquake casualty triage | 40M, leg injury, family missing |
| EP11 | Adult (chronic) | Liver cirrhosis | 62M, ascites, dietary guidance |
| EP12 | Psychiatric | Alcohol dependence | 50M, withdrawal, denial |
| EP13 | Psychiatric | Dementia (BPSD) | 78F, wandering, caregiver burden |
| EP14 | Psychiatric | Adolescent self-harm | 16F, school refusal, trust building |
| EP15 | Geriatric (end-of-life) | Advanced aging — family bereavement | 102F (family: daughter 60s) |
| EP16 | Home-care | Stroke rehabilitation (home visit) | 70M, ADL assessment |
| EP17 | Home-care | Terminal cancer (home hospice) | 68F, pain management |
| EP18 | Adult (acute) | Acute myocardial infarction | 55M, chest pain, anxiety |
| EP19 | Adult (surgical) | Breast cancer (perioperative) | 45F, body image, lymphedema |
| EP20 | Emergency/Disaster | Typhoon power outage ICU | 60M, ventilator anxiety |

## Domain Distribution Summary (All 251 Scenarios)

| **Domain** | **Care plans** | **National exam** | **Total** | **%** |
| --- | --- | --- | --- | --- |
| Acute | 48 | 5 | 53 | 21.1% |
| Chronic | 32 | 2 | 34 | 13.5% |
| Geriatric | 32 | 2 | 34 | 13.5% |
| Pediatric | 24 | 3 | 27 | 10.8% |
| Psychiatric | 32 | 3 | 35 | 13.9% |
| Maternal | 31 | 2 | 33 | 13.1% |
| Home-care | 32 | 2 | 34 | 13.5% |
| Emergency/Disaster | 0 | 1 | 1 | 0.4% |
| Total | 231 | 20 | 251 | 100% |

## Scenario Prompt Structure

Each scenario consists of a paired nurse prompt and patient prompt. The structure is identical across all 251 scenarios:

**Nurse-side system prompt template:**

*You are a [role] nurse. Setting: [clinical scene]. You assess the patient/family with empathy and appropriate clinical judgment. Limit to 1–2 questions/actions per turn; be concise and respectful. Output only the nurse's spoken words. No bracketed descriptions, stage directions, or narration.*

**Patient-side system prompt template:**

*You are a patient. [Age, sex, name]. Chief complaints: [symptoms in lay language]. [Disease-specific behavioral instructions: speech register, affect, family situation]. Output only the patient's spoken words. No bracketed descriptions, stage directions, or narration.*

**Layer-3 individualization:** Per-case clinical details (vital signs, family composition, disease stage, specific concerns) are embedded in the patient prompt and are unique to each of the 251 scenarios.

## Notes

- All scenarios are in Japanese. The table above presents English translations for accessibility.
- The standard nursing care plans are institutional teaching materials authored by the first author; they are not derived from copyrighted textbook prose.
- National-examination items are publicly released by the Japanese Ministry of Health, Labour and Welfare; reconstruction as simulated-patient scenarios falls under Japanese Copyright Act Article 30-4 (use of works for information analysis).
