# Supplementary material for: Coexistence of AI Nurse and AI-Simulated Patient in Nursing Education Using a Bidirectional Fine-Tuning Pipeline: Development and Content Analysis Study
Source: JMIR Biomed Eng. 2026 Nov 6;11:e100778. doi: 10.2196/100778 (PMC13743070; doi:10.2196/100778)
Supplement: Supplementary file 3 [file biomedeng-v11-e100778-s003.docx]

Multimedia Appendix 3

# UI Screenshots and Operating-Mode Walkthrough

## Overview

This appendix provides a walkthrough of the three coexistence operating modes (Mode A, B, C) as implemented in the working web UI. The system runs entirely on a single consumer-grade GPU (NVIDIA RTX 4090) using Ollama for LLM inference, VOICEVOX / AivisSpeech for voice synthesis, and a browser-based frontend.

## System Architecture for UI Deployment

```
┌──────────────────────────────────────────────────┐
│                  Browser (Frontend)               │
│  ┌──────────┐  ┌──────────┐  ┌──────────┐       │
│  │  Mode A   │  │  Mode B   │  │  Mode C   │       │
│  │ AI–AI Demo│  │Student-as │  │Student-as │       │
│  │           │  │  -Nurse   │  │ -Observer │       │
│  └────┬──────┘  └────┬──────┘  └────┬──────┘       │
│       │              │              │               │
└───────┼──────────────┼──────────────┼───────────────┘
        │              │              │
┌───────┼──────────────┼──────────────┼───────────────┐
│       ▼              ▼              ▼               │
│  Local Server (port 8765)                           │
│  ┌─────────────────────────────────────────────┐   │
│  │  Agent-to-Slot Mapping Engine                │   │
│  │  Mode A: NURSE-ONE → Nurse, PATIENT-ONE → Pt│   │
│  │  Mode B: Human → Nurse, PATIENT-ONE → Pt    │   │
│  │         (NURSE-ONE → shadow/feedback)        │   │
│  │  Mode C: Mode A replay + annotation overlay  │   │
│  └─────────────────────────────────────────────┘   │
│                                                     │
│  ┌──────────────┐  ┌──────────────┐                │
│  │ Ollama       │  │ VOICEVOX /   │                │
│  │ NURSE-ONE    │  │ AivisSpeech  │                │
│  │ PATIENT-ONE  │  │ (TTS)        │                │
│  │ (port 11434) │  │ (port 50021) │                │
│  └──────────────┘  └──────────────┘                │
│                                                     │
│  Hardware: RTX 4090 (24 GB VRAM)                   │
│  No cloud dependency                                │
└─────────────────────────────────────────────────────┘
```

## Mode A — AI–AI Demonstration

**Purpose:** Both NURSE-ONE and PATIENT-ONE converse autonomously. The learner observes a complete simulated clinical encounter.

**Agent-to-slot mapping:**

- Nurse slot → NURSE-ONE (GGUF q4_k_m, Ollama)
- Patient slot → PATIENT-ONE (GGUF q4_k_m, Ollama)
- Human role → Observer (no input required)

**UI elements:**

1. **Scenario selector:** Dropdown menu listing all 251 scenarios, organized by domain (7 domains + examination items).
2. **Dialogue panel:** Split-view showing nurse utterances (left, blue background) and patient utterances (right, green background). Each turn is time-stamped with generation latency.
3. **Character display:** Patient character image (generated via ComfyUI) and nurse character image displayed above their respective dialogue columns.
4. **Voice playback:** Each utterance is synthesized via VOICEVOX/AivisSpeech and played sequentially. Audio playback can be paused, replayed, or skipped.
5. **Latency indicator:** Per-turn response time displayed in milliseconds. Median: 1.5–2.9 s (within ≤3 s threshold).
6. **Session controls:** Start / Pause / Reset buttons. maxmsg counter shows remaining turns.

**Pedagogical use:**

- Instructor plays a scenario for a class; students observe and take notes.
- Multiple scenarios can be run consecutively for comparative analysis.
- Dialogue transcripts are downloadable as text files for post-session review.

**Walkthrough:**

7. Select scenario (e.g., "Heart Failure — 75-year-old male, Sato-san")
8. Click "Start Dialogue"
9. NURSE-ONE generates first turn → voice plays → PATIENT-ONE responds → voice plays → cycle continues
10. Dialogue ends at maxmsg=25 or when clinical endpoint is reached
11. Full transcript is displayed; instructor can highlight specific turns for discussion

## Mode B — Student-as-Nurse

**Purpose:** The learner replaces NURSE-ONE and engages directly with PATIENT-ONE. NURSE-ONE provides scaffolding in one of two sub-modes.

**Agent-to-slot mapping:**

- Nurse slot → Human learner (text input)
- Patient slot → PATIENT-ONE (GGUF q4_k_m, Ollama)
- NURSE-ONE role → Shadow reference (B1) or Post-hoc feedback (B2)

### Mode B1 — Reference Mode

**UI elements:**

12. **Student input panel:** Text input field where the student types their nurse response.
13. **Patient response panel:** PATIENT-ONE's response appears after the student submits.
14. **NURSE-ONE shadow panel:** After each student turn, NURSE-ONE generates what it *would have said* in the same situation. Displayed in a collapsible sidebar labeled "Reference: NURSE-ONE's response" (light gray background).
15. **Comparison view:** Student's utterance and NURSE-ONE's utterance displayed side-by-side for self-assessment.

**Walkthrough:**

16. Select scenario → Click "Start (B1: Reference Mode)"
17. PATIENT-ONE introduces chief complaint (first turn)
18. Student types nurse response → submits
19. System simultaneously: (a) sends student's text to PATIENT-ONE for next patient turn, (b) sends the same context to NURSE-ONE for shadow generation
20. Both responses appear: patient's reply to the student, and NURSE-ONE's alternative nurse response (collapsible)
21. Student can compare their approach with NURSE-ONE's at each turn
22. Process repeats until session ends

### Mode B2 — Feedback Mode

**UI elements:**

23. **Student input panel:** Same as B1.
24. **Patient response panel:** Same as B1.
25. **No shadow panel during encounter:** NURSE-ONE is withheld during the live interaction.
26. **Post-hoc feedback panel:** After the encounter ends, NURSE-ONE receives the complete student utterance sequence and generates structured commentary including: (a) strengths identified, (b) missed assessment opportunities, (c) communication-style observations, (d) Berelson-category comparison with NURSE-ONE's expected pattern.

**Walkthrough:**

27. Select scenario → Click "Start (B2: Feedback Mode)"
28. Student interacts with PATIENT-ONE without any NURSE-ONE reference
29. Encounter concludes (maxmsg or clinical endpoint)
30. System sends full student transcript to NURSE-ONE with a feedback prompt
31. Structured feedback appears in a dedicated panel
32. Student reviews feedback alongside their own transcript

## Mode C — Student-as-Observer

**Purpose:** The learner reviews a pre-recorded Mode A dialogue and annotates it using the Berelson schema or a custom rubric.

**Agent-to-slot mapping:**

- Nurse slot → NURSE-ONE (pre-recorded Mode A dialogue)
- Patient slot → PATIENT-ONE (pre-recorded Mode A dialogue)
- Human role → Annotator (labeling each turn)

**UI elements:**

33. **Dialogue replay panel:** Mode A dialogue displayed turn-by-turn.
34. **Annotation overlay:** For each nurse turn, a dropdown menu with the six Berelson categories (OQ, CQ, IN, AC, EM, OT). Learner selects the category they believe applies.
35. **Custom rubric option:** Instructor can define alternative category sets (e.g., therapeutic communication techniques, clinical reasoning markers).
36. **Scoring summary:** After annotation, a comparison table shows: student's labels, instructor's reference labels (if pre-entered), and agreement percentage.
37. **Multi-observer support:** Multiple students can annotate the same dialogue independently; cross-observer agreement (κ) can be computed for class discussion.

**Walkthrough:**

38. Instructor selects a pre-recorded Mode A dialogue
39. Click "Start Annotation (Mode C)"
40. Dialogue is displayed turn-by-turn; after each nurse turn, student selects Berelson category
41. After completing all turns, student submits annotations
42. System displays comparison with reference annotations and calculates agreement
43. Instructor-led discussion of disagreements and category-boundary cases

## Latency Performance Summary

| Mode | Agent turns per session | Median latency per turn | Meets ≤3 s threshold? |
| --- | --- | --- | --- |
| Mode A (AI–AI) | ~25 (both agents) | 2.5–2.9 s | ✅ Yes |
| Mode B1 (Reference) | ~12 student + ~12 PATIENT-ONE + ~12 NURSE-ONE shadow | 1.5–2.0 s (PATIENT-ONE); 1.5–2.0 s (shadow) | ✅ Yes |
| Mode B2 (Feedback) | ~12 student + ~12 PATIENT-ONE + 1 feedback | 1.5–2.0 s (PATIENT-ONE); ~5–8 s (feedback, single generation) | ✅ Yes (per-turn) |
| Mode C (Observer) | 0 (replay) | N/A (pre-recorded) | ✅ Yes |

All per-turn latencies are for text generation only (Ollama inference on RTX 4090 with GGUF q4_k_m models). Voice synthesis (VOICEVOX) adds approximately 0.5–1.0 s per utterance.

## Offline Deployment Configuration

The system is designed for complete offline operation:

| Component | Software | Network requirement |
| --- | --- | --- |
| LLM inference | Ollama (local) | None |
| Voice synthesis | VOICEVOX (local, port 50021) | None |
| Frontend | Browser (localhost:8765) | None |
| Model files | GGUF q4_k_m on local SSD | None |

**Total storage requirement:** ~12 GB (NURSE-ONE 5.07 GB + PATIENT-ONE 5.07 GB + VOICEVOX models ~2 GB)

**Hardware requirement:** NVIDIA RTX 4090 (24 GB VRAM) or equivalent; Windows 11

This configuration supports disaster-preparedness deployment: the system operates without internet connectivity, cloud APIs, or external servers.

## Notes

- UI screenshots referenced in this appendix correspond to the working web UI implementation. Full-resolution screenshots are available from the corresponding author.
- The UI design is functional (research prototype); production-quality UI design is identified as future work.
- All scenario content, model weights, and voice-synthesis parameters are stored locally; no data leaves the device during operation.
