# Supplementary material for: Coexistence of AI Nurse and AI-Simulated Patient in Nursing Education Using a Bidirectional Fine-Tuning Pipeline: Development and Content Analysis Study
Source: JMIR Biomed Eng. 2026 Nov 6;11:e100778. doi: 10.2196/100778 (PMC13743070; doi:10.2196/100778)
Supplement: Supplementary file 4 [file biomedeng-v11-e100778-s004.docx]

# Multimedia Appendix 4

## Human Coder Validation Data for Berelson Content Analysis

Two independent nursing experts (Coder A and Coder C) classified a stratified subsample of nurse-side utterances using the same six-category Berelson scheme applied by the LLM raters. Sampling, blinding, and results are summarized below.

### Sampling Procedure

From each of the four training-volume conditions (base, x1/245, x3/735, x5/1225), 50 utterances were drawn via stratified random sampling (seed = 20260721) preserving category proportions from the full 200-utterance pool. Total sample: 4 conditions x 50 = 200 utterances. Condition identifiers were randomized to Set A-D (blinding key: A = base, B = x5, C = x1, D = x3). Four utterances from Coder A were missing, yielding n = 196 for analysis.

### Overall Inter-Rater Reliability

| **Measure** | **Value** |
| --- | --- |
| Cohen's kappa | 0.770 |
| Percent agreement | 82.7% |
| n | 196 |
| Interpretation (Landis & Koch 1977) | Substantial agreement |

### Condition-Level Kappa

| **Condition** | **Kappa** | **Agreement** | **n** |
| --- | --- | --- | --- |
| Base (no training) | 0.645 | 73.5% | 49 |
| x1 (245 cases) | 0.778 | 83.7% | 49 |
| x3 (735 cases) = NURSE-ONE | 0.809 | 85.7% | 49 |
| x5 (1225 cases) | 0.839 | 87.8% | 49 |

### Category-Level Agreement

| **Category** | **Agreements** | **Total** | **Agreement %** |
| --- | --- | --- | --- |
| OQ (Open Question) | 35 | 46 | 76.1% |
| CQ (Closed Question) | 19 | 33 | 57.6% |
| EM (Empathy/Acceptance) | 30 | 46 | 65.2% |
| IN (Information Collection) | 12 | 14 | 85.7% |
| AC (Assessment Confirmation) | 65 | 85 | 76.5% |
| OT (Other) | 1 | 6 | 16.7% |

### Comparison With LLM Inter-Rater Reliability

| **Rater Pair** | **Kappa** | **Interpretation** |
| --- | --- | --- |
| Human coders (A x C) | 0.770 | Substantial |
| LLM raters (Sonnet 4.5 x Opus 4.6) | 0.742 | Substantial |

### Notes

Coder B (a nurse who had left clinical practice) was excluded due to low inter-coder agreement with other coders (kappa = 0.44-0.47), attributable to idiosyncratic classification criteria.

LLM labels were generated by Claude Sonnet 4.5 (2026-04-21). Human coders received identical six-category definitions and classification rules.

## Round 2 Extended Validation: Full-Dataset Human Coding (Response to Reviewer Comment E3)

In response to Reviewer AI’s Round 2 comment (E3) that the stratified-subsample validation reported above was insufficient, two additional independent nursing experts (Coder D and Coder E, distinct from Coders A–C used in the Round 1 validation above) classified the full dataset used in the main Berelson analyses — all 1,520 utterances (800 nurse-side utterances across the four training-volume conditions and 720 patient-side utterances across the A/B/C conditions) — rather than a stratified subsample. To increase classification rigor, each coder first wrote a brief free-text interpretation of the utterance’s communicative intent before assigning a Berelson category, reducing reliance on surface-level pattern matching.

### Overall Inter-Rater Reliability (Full Dataset)

| Measure | Value |
| --- | --- |
| Cohen's kappa | 0.763 |
| Percent agreement | 82.5% |
| n | 1,520 |
| Interpretation (Landis & Koch 1977) | Substantial agreement |

### Category Distribution by Coder

| Category | Coder D, n (%) | Coder E, n (%) | Difference |
| --- | --- | --- | --- |
| AC | 393 (25.9%) | 331 (21.8%) | -62 |
| CQ | 164 (10.8%) | 305 (20.1%) | +141 |
| EM | 114 (7.5%) | 99 (6.5%) | -15 |
| IN | 619 (40.7%) | 610 (40.1%) | -9 |
| OQ | 211 (13.9%) | 155 (10.2%) | -56 |
| OT | 19 (1.3%) | 20 (1.3%) | +1 |

### Notes on Round 2 Extended Validation

The interpretation-first classification procedure yielded substantially higher agreement (kappa = 0.763) than the direct classification used in the Round 1 subsample validation and in a preliminary Round 2 pass without interpretation notes (kappa = 0.580, not reported in the main text). The main residual disagreement was in the CQ (closed question) category, where Coder E classified more utterances as CQ (305) than Coder D (164); inspection of the confusion pattern showed most of this difference was drawn from utterances Coder D classified as OQ (52 cases) or AC (96 cases), suggesting a boundary disagreement on questions that combine an open-ended invitation with an embedded closed-form check, rather than a systematic coder-specific bias.
