# Supplementary material for: Coexistence of AI Nurse and AI-Simulated Patient in Nursing Education Using a Bidirectional Fine-Tuning Pipeline: Development and Content Analysis Study
Source: JMIR Biomed Eng. 2026 Nov 6;11:e100778. doi: 10.2196/100778 (PMC13743070; doi:10.2196/100778)
Supplement: Supplementary file 5 [file biomedeng-v11-e100778-s005.docx]

# Multimedia Appendix 5

## Patient-Side A/B/C Statistical Comparison (Response Length and Category Distribution)

This appendix reports the full statistical comparison underlying the selection of condition C (735 training cases) as the production PATIENT-ONE model, prepared in response to reviewer comment AI-R2-2 (Round 2). All analyses use the full patient-side Berelson-labeled dataset (n=240 utterances per condition, 720 total).

### Full Category Distribution

| Condition | Training cases | Response length, mean (SD) | AC | CQ | EM | IN | OQ/OT |
| --- | --- | --- | --- | --- | --- | --- | --- |
| A (base) | 0 | 76.1 (29.8) | 4.2% | 0.4% | 32.5% | 43.3% | OQ 5.0% / OT 14.6% |
| B (prototype patient 1) | 245 | 51.1 (27.2) | 8.3% | 1.7% | 26.2% | 40.4% | OQ 5.4% / OT 17.9% |
| C (prototype patient 2, adopted as PATIENT-ONE) | 735 | 51.1 (24.5) | 4.2% | 1.2% | 27.9% | 47.9% | OQ 3.8% / OT 15.0% |

### Response-Length Statistical Tests

One-way ANOVA across the three conditions confirmed a significant overall difference (F(2,717)=67.198, p=1.79e-27, eta-squared=0.158, a large effect).

| Pairwise comparison | Welch's t | p (raw) | Cohen's d |
| --- | --- | --- | --- |
| A vs B | 9.587 | 5.13e-20 | 0.875 |
| A vs C | 10.037 | 1.43e-21 | 0.916 |
| B vs C | 0.004 | 0.997 | 0.000 |

Bonferroni-corrected alpha for three pairwise comparisons = 0.0167. A vs B and A vs C are significant at this threshold; B vs C is not (p=0.997), indicating that fine-tuning per se (A to B/C) drives the length reduction, while the further difference between B and C is not statistically distinguishable from zero.

### Levene's Test for Equality of Variance

| Comparison | W | p |
| --- | --- | --- |
| All three conditions | 2.130 | 0.120 |
| A vs B | 0.602 | 0.438 |
| A vs C | 3.708 | 0.055 |
| B vs C | 2.243 | 0.135 |

No pairwise or omnibus comparison reached significance, indicating that the numerically lower SD in condition C (24.5 vs. 27.2 in condition B) is not a statistically robust variance reduction.

### Selection Rationale

Condition C was selected as the production PATIENT-ONE on the basis of its marginally higher IN rate (47.9% vs. 40.4% for condition B) and marginally lower response-length SD (24.5 vs. 27.2), consistent with the exploratory (post-hoc) rather than confirmatory framing of the A/B/C comparison reported in the main text. No pre-specified statistical threshold for this selection was defined prior to observing the three conditions' results.
