# Supplementary material for: Coexistence of AI Nurse and AI-Simulated Patient in Nursing Education Using a Bidirectional Fine-Tuning Pipeline: Development and Content Analysis Study
Source: JMIR Biomed Eng. 2026 Nov 6;11:e100778. doi: 10.2196/100778 (PMC13743070; doi:10.2196/100778)
Supplement: Supplementary file 6 [file biomedeng-v11-e100778-s006.docx]

Multimedia Appendix 6

# Sample Multi-Turn Dialogues from Each of the Seven Nursing Domains (Mode A: AI–AI Demonstration)

## Overview

This appendix presents sample dialogues from Mode A (AI–AI demonstration), in which NURSE-ONE and PATIENT-ONE converse without human intervention. One representative scenario per domain is shown. All dialogues are in Japanese (the system's operating language) with English translations provided in brackets. Dialogues were collected using AutoGen v18 with maxmsg=25; only the initial turns are shown here for space.

## Domain 1: Acute Care — Heart Failure (75-year-old male, Mr. Sato)

**Scenario:** Outpatient initial assessment. One week of worsening dyspnea on exertion, orthopnea, pedal edema, 3 kg weight gain. History of myocardial infarction 10 years ago; on antihypertensives. Lives with wife.

| Turn | Role | Utterance (Japanese) | English translation |
| --- | --- | --- | --- |
| 1 | NURSE-ONE | 佐藤さん、今日はどれくらい息苦しいですか？特に夜間が一番つらいとのことですが、具体的にどんな感じですか？ | Mr. Sato, how breathless are you feeling today? You mentioned nighttime is the worst — can you describe what it's like specifically? |
| 2 | PATIENT-ONE | うーん…夜中に横になってると、だんだん胸が苦しくなってきて…起き上がると少し楽になるんだけど、またすぐ苦しくなるんです。 | Hmm… when I lie down at night, my chest gradually gets tight… sitting up helps a bit, but then it gets bad again soon. |
| 3 | NURSE-ONE | 起き上がると少し楽になるんですね。枕を高くしたりしていますか？それから、足のむくみはいつ頃から気になり始めましたか？ | So sitting up relieves it somewhat. Have you tried raising your pillows? And when did you first notice the swelling in your legs? |
| 4 | PATIENT-ONE | 枕は二つ重ねてるんだけど…足のむくみは、一週間くらい前からかな。靴下の跡がくっきり残るようになって。 | I've been stacking two pillows… The leg swelling, maybe about a week ago. The sock marks started staying really visible. |

**Berelson classification of NURSE-ONE turns:** Turn 1 = OQ (open question); Turn 3 = CQ + IN (closed question on pillows, information collection on edema onset).

## Domain 2: Chronic Care — Type 2 Diabetes (55-year-old male, Mr. Yamada)

**Scenario:** Outpatient initial assessment. Polydipsia, nocturia, weight loss. HbA1c elevated but neglected. Sedentary, high-fat diet. Resistant to lifestyle change.

| Turn | Role | Utterance (Japanese) | English translation |
| --- | --- | --- | --- |
| 1 | NURSE-ONE | 最近、喉の渇きや夜間の排尿回数の増加、体重減少など、複数の症状があるようですね。生活習慣や食生活についても詳しくお伺いしてもよろしいでしょうか。 | It sounds like you've been experiencing several symptoms — increased thirst, more nighttime urination, and weight loss. May I ask you in detail about your daily habits and diet? |
| 2 | PATIENT-ONE | ああ、まあ…のどが渇くのは暑いからでしょ。トイレの回数は、年のせいじゃないですか。体重は…確かに少し減ったけど、別にダイエットしたわけじゃないし。 | Ah, well… the thirst is just because it's hot, right? The bathroom trips — isn't that just getting older? My weight, sure it went down a bit, but it's not like I was dieting or anything. |
| 3 | NURSE-ONE | なるほど、そうお感じなんですね。ところで、普段のお食事はどんな感じですか？例えば、揚げ物やジュースはお好きですか？ | I see, that's how you feel about it. By the way, what are your usual meals like? For example, do you enjoy fried food or juice? |
| 4 | PATIENT-ONE | 揚げ物は好きですよ、毎日とは言わないけど。ジュースも…コーラとか、仕事中に飲んじゃいますね。忙しいんで、なかなかね。 | I do like fried food, not every day but… And juice — I drink cola and stuff at work. I'm busy, so it's hard, you know. |

**Berelson classification of NURSE-ONE turns:** Turn 1 = IN (information collection on lifestyle); Turn 3 = CQ (closed question on specific dietary items).

## Domain 3: Geriatric Care — Early Dementia (82-year-old female, Mrs. Takahashi)

**Scenario:** Outpatient initial assessment. Increasing forgetfulness, repeating stories, burning pots, forgetting appointments. Denies problems ("it's just age"). Accompanied by daughter.

| Turn | Role | Utterance (Japanese) | English translation |
| --- | --- | --- | --- |
| 1 | NURSE-ONE | お疲れ様でした。今日はどうされましたか？最近何か気になることはありますか？ | Thank you for coming. What brings you in today? Is there anything that's been on your mind recently? |
| 2 | PATIENT-ONE | ああ、別に何ともないですよ。娘がね、心配性で連れてきたんですよ。私はぴんぴんしてますから。 | Oh, there's nothing wrong with me at all. My daughter is such a worrier — she dragged me here. I'm perfectly fine. |
| 3 | NURSE-ONE | そうですか、お元気そうで何よりです。最近、お料理やお買い物はいつもどおりできていますか？ | I see, I'm glad you seem well. Have you been able to cook and shop as usual lately? |
| 4 | PATIENT-ONE | もちろんですよ。毎日ちゃんとご飯作ってますから。…あ、この前ちょっと鍋をね、焦がしちゃったけど、あれはたまたまですよ。 | Of course. I cook every day. …Oh, I did burn a pot the other day, but that was just a fluke. |

**Berelson classification of NURSE-ONE turns:** Turn 1 = OQ (open question); Turn 3 = CQ (closed question on specific ADL).

## Domain 4: Pediatric Care — Childhood Leukemia (8-year-old boy, Sato-kun)

**Scenario:** Ward round. ALL, fever since yesterday, nausea. Lonely about missing school friends. Speaks honestly but in a childlike, dependent manner.

| Turn | Role | Utterance (Japanese) | English translation |
| --- | --- | --- | --- |
| 1 | NURSE-ONE | 佐藤くん、おはよう。昨日の夜はよく眠れた？今日の体の調子はどうかな？ | Good morning, Sato-kun. Did you sleep well last night? How are you feeling today? |
| 2 | PATIENT-ONE | あー、なんかお腹が痛いし、熱っぽいし…。気持ち悪いよぉ。 | Ugh, my tummy hurts and I feel hot… I feel sick. |
| 3 | NURSE-ONE | そうかぁ、つらいね。熱っぽいのはいつからかな？お腹はどこが一番痛い？ | Oh, that sounds tough. When did you start feeling hot? Where does your tummy hurt the most? |
| 4 | PATIENT-ONE | きのうの夜からだよ…。お腹はね、ここらへん…全体的にムカムカする感じ。 | Since last night… My tummy, around here… it's like a general queasy feeling all over. |

**Berelson classification of NURSE-ONE turns:** Turn 1 = OQ + CQ (open question on condition, closed question on sleep); Turn 3 = IN (information collection on onset and location).

## Domain 5: Psychiatric Care — Depression (42-year-old female, Ms. Kimura)

**Scenario:** Outpatient visit. Depression triggered by workplace stress. Chief complaints: insomnia for 2 weeks, unable to get out of bed, suicidal ideation ("it would be better to be dead"). Soft voice, flat affect.

| Turn | Role | Utterance (Japanese) | English translation |
| --- | --- | --- | --- |
| 1 | NURSE-ONE | 木村さん、本日はお疲れ様でした。まず、今一番つらいのは眠れないことですか？ | Ms. Kimura, thank you for coming today. First, is the hardest thing right now the inability to sleep? |
| 2 | PATIENT-ONE | …はい。もう2週間くらい…ほとんど眠れなくて。布団に入っても、ぐるぐる考えてしまって…。 | …Yes. It's been about 2 weeks… I can barely sleep at all. Even in bed, my thoughts keep going round and round… |
| 3 | NURSE-ONE | 2週間も眠れないのはとてもつらいですね。考え事の内容は、お仕事のことが多いですか？ | Not sleeping for 2 weeks must be very hard. Are the things you keep thinking about mostly work-related? |
| 4 | PATIENT-ONE | 仕事のことも…でも、それだけじゃなくて…自分がダメな人間なんじゃないかって…。 | Work things too… but not just that… I keep thinking maybe I'm a worthless person… |

**Berelson classification of NURSE-ONE turns:** Turn 1 = CQ (closed question confirming chief complaint priority); Turn 3 = EM + CQ (empathic acknowledgement followed by closed question on thought content).

## Domain 6: Maternal Care — Pregnancy-Induced Hypertension (34-year-old female, Ms. Ito)

**Scenario:** Admission assessment. 34 weeks gestation, second pregnancy. Facial and pedal edema, headache, high blood pressure. First pregnancy was normal; confused and anxious about this complication. Worried about preterm delivery and fetal impact.

| Turn | Role | Utterance (Japanese) | English translation |
| --- | --- | --- | --- |
| 1 | NURSE-ONE | 伊藤さん、おはようございます。まず今の体調から確認させてください。頭痛の程度はいかがですか？ | Good morning, Ms. Ito. Let me start by checking how you're feeling. How is the headache? |
| 2 | PATIENT-ONE | 頭がズキズキして…朝起きたら顔がすごくむくんでて、足もパンパンで…。血圧が高いって言われてすごく怖いんです。 | My head is throbbing… When I woke up, my face was really swollen, and my legs are puffy… I'm so scared because they said my blood pressure is high. |
| 3 | NURSE-ONE | 不安ですよね。前回のご出産は問題なかったとのことで、今回のことに戸惑われていると思います。赤ちゃんの胎動は感じていますか？ | It must be frightening. Since your first delivery went smoothly, I imagine this is confusing. Are you feeling the baby's movements? |
| 4 | PATIENT-ONE | 胎動は…あります。でも前回と違って、こんなことになるなんて…。赤ちゃんに何かあったらどうしようって、ずっと考えてしまって…。 | The baby is moving… yes. But unlike last time, I never expected this… I keep worrying, what if something happens to the baby… |

**Berelson classification of NURSE-ONE turns:** Turn 1 = CQ (closed question on headache severity); Turn 3 = EM + CQ (empathic acknowledgement of anxiety, closed question on fetal movement).

## Domain 7: Home-Care — ALS (58-year-old male, Mr. Shimizu)

**Scenario:** Home-visit nursing assessment. ALS diagnosed 2 years ago, wife as primary caregiver. Nocturnal dyspnea worsening, dysarthria progressing, fear of losing toilet independence. Former engineer, logical thinker. Speaks quietly, with difficulty.

| Turn | Role | Utterance (Japanese) | English translation |
| --- | --- | --- | --- |
| 1 | NURSE-ONE | 清水さん、今日はお邪魔します。最近の呼吸の具合はいかがですか？特に夜間の息苦しさについて教えてください。 | Mr. Shimizu, thank you for having me today. How has your breathing been recently? Please tell me especially about the nighttime breathlessness. |
| 2 | PATIENT-ONE | …夜中にね、急に息が…苦しくなって。横になってると…だめで。 | …At night, suddenly I can't… breathe. Lying down… doesn't work. |
| 3 | NURSE-ONE | 夜中に急に息苦しくなるのですね。痰が絡む感じはありますか？それと、日中の活動で息切れが強くなる場面はありますか？ | So you get suddenly breathless at night. Do you feel phlegm building up? And are there daytime activities that make the breathlessness worse? |
| 4 | PATIENT-ONE | 痰は…ある。自分で出せなくなってきた…。トイレに行くだけで…息が切れて。 | Phlegm… yes. I'm losing the ability to clear it myself… Just going to the toilet… leaves me breathless. |

**Berelson classification of NURSE-ONE turns:** Turn 1 = OQ (open question on respiratory status); Turn 3 = CQ + IN (closed question on phlegm, information collection on exertional dyspnea).

## Notes

- All dialogues shown here are AI–AI interactions (Mode A); no human subjects participated.
- Dialogue texts are direct outputs from NURSE-ONE and PATIENT-ONE running on Ollama (GGUF q4_k_m) on a single RTX 4090. No post-editing was applied.
- English translations are provided for the convenience of non-Japanese-reading reviewers. The system operates exclusively in Japanese.
- Full-length dialogue transcripts (maxmsg=25 per session) across all 251 scenarios are available from the corresponding author upon request.
